# Fine-Root Traits Reveal Contrasting Ecological Strategies in European Beech and Norway Spruce During Extreme Drought

**DOI:** 10.3389/fpls.2020.01211

**Published:** 2020-08-13

**Authors:** Petia Simeonova Nikolova, Taryn L. Bauerle, Karl-Heinz Häberle, Helmut Blaschke, Ivano Brunner, Rainer Matyssek

**Affiliations:** ^1^Forest Resources and Management, Swiss Federal Research Institute WSL, Birmensdorf, Switzerland; ^2^Ecophysiology of Plants, TUM School of Life Sciences, Technische Universität München, Freising, Germany; ^3^School of Integrative Plant Science, Cornell University, Ithaca, NY, United States; ^4^Forest Soils and Biogeochemistry, Swiss Federal Research Institute WSL, Birmensdorf, Switzerland

**Keywords:** ecological strategy, extreme drought, *Fagus sylvatica*, fine-root classification, fine-root traits, morphology, non-structural carbohydrates, *Picea abies*

## Abstract

Trees adjust multiple structural and functional organ-specific characteristics, “traits”, to cope with diverse soil conditions. Studies on traits are widely used to uncover ecological species adaptability to varying environments. However, fine-root traits are rarely studied for methodological reasons. We analyzed the adaptability of the fine-root systems of European beech and Norway spruce to extreme drought within species-specific tree groups at Kranzberger Forst (Germany), focusing on the seasonality of morphological, physiological, and biochemical key traits in view of carbon (C) and nitrogen dynamics. We hypothesized that fine roots of both species adjust to seasonal drought: with beech representing a “fast” (i.e. with fast C turnover), and spruce a “slow” (i.e. with long-term C retention) ecological strategy. We identified three functional fine-root categories, based on root function (absorptive or transport fine roots), and mycorrhizal status of the absorptive fine-roots (mycorrhizal or non-mycorrhizal). Solely the non-mycorrhizal absorptive roots adjusted in a species-specific manner supporting fine-root ecological strategy hypothesis. During drought, beech produced thin ephemeral (absorptive non-mycorrhizal) fine roots with high specific fine-root area and high respiratory activity, representing fast C turnover and enabling effective resource exploitation. These adjustments reflect a “fast” ecological strategy. Conversely, spruce absorptive fine roots did not respond to the soil moisture deficit by growth but instead increased root suberization. Drastically lowered respiratory activity of this functional category facilitated C retention and structural persistence during drought, indicating a “slow” ecological strategy in spruce. Absorptive mycorrhizal fine roots maintained respiration throughout the drought event in both tree species, but in spruce this was the only fine-root category with high respiration. This suggests, that spruce relies heavily on mycorrhizal associations as a method of drought resistance. Accumulation of non-structural carbohydrates and high C concentrations were observed in the transport fine roots of both species, indicating drought-induced osmotic protection of these roots. Thus, functional classification enabled us to determine that fine-root branches of each species are not tied to one sole ecological strategy. The suggested approach helps to better understand the complex interplay between structure and function belowground.

## Introduction

Plants and especially long-lived trees have evolved a variety of structural and functional characteristics (“traits”) both above- and belowground to optimize the use of heterogeneous spatiotemporal resources (Lavorel and Garnier, 2002; Rennenberg et al., 2006; Freschet et al., 2018). Analyses of plant traits make it possible to decipher species-specific trade-offs in adapting to resource limited site conditions (Iversen et al., 2017; McCormack et al., 2017; Brunner et al., 2019). Belowground traits include root morphology and physiology along with mycorrhizal associations in relation to seasonal and soil variation (Laliberté, 2017). Although more than 300 root traits have been identified across individual studies (Iversen et al., 2017), they remain underrepresented in global trait databases (Ma et al., 2018). Frequently, the lack of root trait data is a direct result of methodological sampling difficulty (Joslin et al., 2000; Pregitzer, 2002; Brunner et al., 2015), challenges in integrating outcomes from varying environments, diverse measurement techniques and complex species-specific stress responses (Iversen et al., 2017).

Fine roots, commonly defined as <2 mm in diameter (Böhm, 1979), are the most physiologically active plant components of a root system. Shifts in fine-root diameter can serve as a proxy for root water/nutrient uptake capacity (Zobel et al., 2006; Tobner et al., 2013). However, studies often fail to determine diameter thresholds that indicate changes in root function but, instead, tend to rely on arbitrary thresholds. The thinnest fine-root fraction (e.g., < 1 mm) can better reflect belowground adjustments to resource availability (Leuschner et al., 2001; Zobel et al., 2007). Such thin fine-root laterals, typical of many deciduous tree species, sometimes referred to as fibrous or feeder roots (Sutton and Tinus, 1983), do not undergo secondary growth, are short-lived, and display high N concentration and respiration rate (RR). These laterals are ephemeral, turning over at rates similar to deciduous leaves (Eissenstat et al., 2013). Thin highly ephemeral roots typify a “fast-strategy” and represent one extreme of the whole-plant economic spectrum (Reich, 2014, but see also Withington et al., 2006). While, coarse, slow-growing fine-roots exemplifies a “slow-strategy” (Wang et al., 2016; Leuschner and Meier, 2018). Perennial plants with fine-roots of similar diameter may differ in form and function, which makes diameter-based root trait and biomass data difﬁcult to interpret (Iversen et al., 2017). Therefore, an alternative classification based on defined functional groups provides an improved alternative when comparing across species and sites (Freschet and Roumet, 2017).

The “fast-slow” plant economics spectrum defined by Reich (2014) depicts a range of adaptive organ-specific strategies as basic ecological features of plant life forms. According to Reich (2014), plant organs, i.e. roots and shoots of an individual species, should conform to a resource use strategy with implications for whole-plant performance and community assembly (but see Tobner et al., 2013). Species with rapid resource turnover, so-called “fast” species (Reich, 2014), produce short-lived organs for rapid resource capture and translocation. “Slow” species, however, have long-lived organs but prolonged retention of resources. In the context of roots, “slow” species should possess long-lived fine-roots, low specific fine-root length (SRL, among other related traits) and strongly rely on ectomycorrhizal associations (ECM) (Agerer, 2001; Brundrett, 2002; Bergmann et al., 2020).

We aimed to determine if fine-root strategies of adult beech and spruce trees follow similar (“fast” vs. “slow”) patterns as those found in the leaf economics spectrum. This study utilized the prolonged, extraordinarily hot and dry, summer conditions that prevailed over wide regions of Western and Central Europe in 2003 (Rebetez et al., 2006) including the study site, Kranzberger Forst (Freising, Germany) (Raspe et al., 2004). Fine-root production and fine-root recovery rate were unaffected in beech during drought in 2003 (Nikolova et al., 2009). In contrast, fine root production and biomass recovery rate decreased by almost a factor of six in spruce during the drought year. We therefore hypothesized that beech and spruce represent belowground “fast” and “slow” plant strategies with corresponding fine-root traits. To this end, we examined the fine-root carbon (C) and nitrogen (N) status, morphological parameters, [fine root diameter (D), specific fine-root area (SRA)], and physiological parameters, [fine-root RR and concentration of non-structural carbohydrates (NSC)] in response to seasonal drought. Fine-root samples were classified into three categories, based on fine-root function and mycorrhizal abundance. This classification enabled (1) the quantification of seasonal progressive drought responses within functionally defined fine-root classes, and (2) an *in situ* comparison of fine-root traits between beech and spruce to distinguish underlying mechanisms of belowground drought adaptation.

## Material and Methods

### Site Conditions and Climate

The study was conducted at a mixed European beech-Norway spruce (*Fagus sylvatica* L./*Picea abies* [L.] H. Karst) stand at Kranzberger Forst near Freising, Germany in 2003 (Matyssek et al., 2010). The site is composed of two groups of approximately 70-year-old beech trees, each surrounded by spruce trees, which were taller although younger by about 20 years (Pretzsch et al., 2010; Häberle et al., 2012). Rooting depth was about 1 m in a Luvisol (FAO classification) which had developed from Loess over Tertiary sediments and limited to approximately 1 mby a compacted hardpan layer. Litter layer depth was about 5 cm under the spruce canopy and 3 cm under the beech. The C:N ratio within the upper 10 cm of soil ranged between 14 and 17, with the highest values occurring under beech (Schuhbäck, 2004). Soil nutrients and water were non-limiting during average growth years.

**Figure 1:**
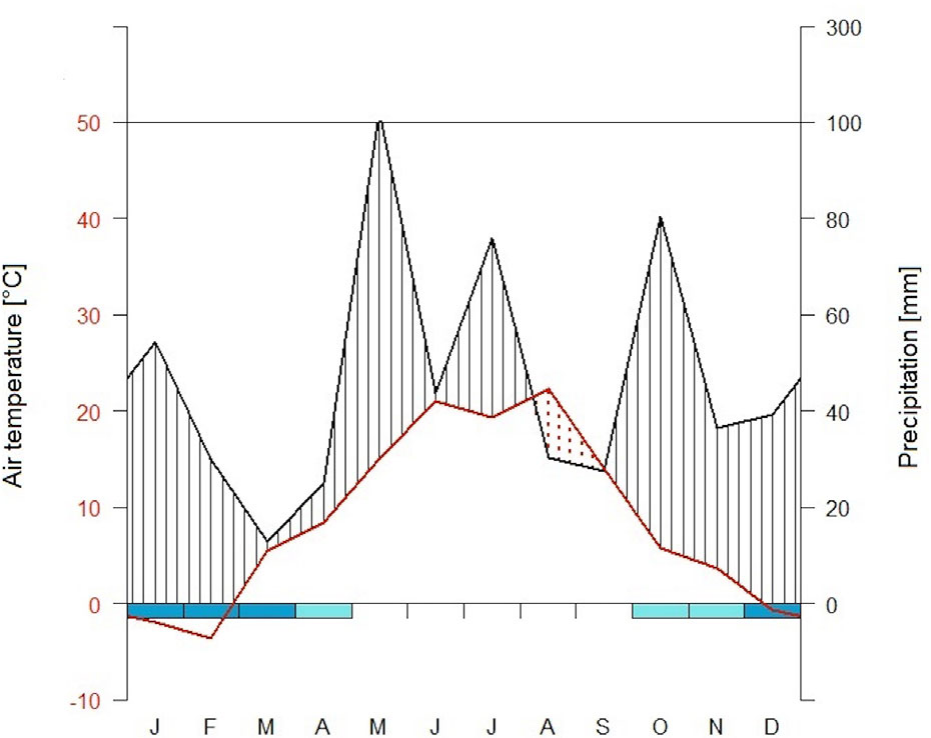
Climate diagram at Kranzberger Forst during the study year of 2003. Scales of air temperature T (red line) and precipitation P (black line) according to Walter and Lieth (1960), i.e. 10°C correspond to 20 mm, respectively. Arid period (when P < 2×T) is filled in dotted red vertical lines, wet periods are filled in black lines. Cold months (when absolute daily minimums ≤ 0°C) are shown in dark blue, probable frost months (when absolute monthly minimums ≤ 0°C) are shown in light blue [using the R package climatol (Guijarro, 2020)].

The study site is classified as temperate based on a 30-year record (1971–2000) of mean daily air temperature (T) and annual precipitation (P) (7.8°C and 786 mm, respectively), with periods of snow cover between December and February (Nikolova et al., 2009). During the 2003 growing season, extreme weather conditions were recorded at Kranzberger Forst (Nikolova et al., 2009): mean T was 3.2 °C higher, and P was 30% lower relative to the long-term seasonal averages. In the same year, a drought period occurred from August through September (**Figure 1**), imposing tree water limitations during the late summer. Soil moisture differed between the two tree species starting in the spring of 2003 (Nikolova et al., 2009), a direct result of the ability of spruce to take up and transpire water before beech flushed its leaves (Beier, 1998). Available soil water was completely depleted in 2003 under spruce by mid-July, under beech by mid-August, respectively. This resulted in a longer period of exhausted soil water availability for spruce (i.e., 75 d in spruce vs. 45 d in beech). Additionally, soil temperature (T_(0)_) was monitored at 0 cm soil depth, i.e. at the border of the humus layer and mineral soil.

### Fine-Root Categories and Sampling

Measurements of fine-root parameters were organized in four sampling campaigns: spring (April, May), early summer (June, July) with exhausted soil water only under spruce, late summer (August) with exhausted soil water under beech and spruce, and autumn (October, November) when available soil water partially recovered under both tree species (Nikolova et al., 2009).

To characterize fine-root trait differences between beech and spruce, three functional fine-root categories were distinguished among sampled fine-root branches that reflect the commonly used < 2 mm in diameter classification, further called “rootlets” based on (1) root function (absorptive or transport fine roots), and (2) mycorrhizal status of the absorptive fine roots (mycorrhizal or non-mycorrhizal) (**Figure S1**):

- Absorptive foraging fine roots (FR): fast-growing, non-mycorrhizal fine roots with primary xylem, primarily serving for soil exploration (Guo et al., 2008; Zadworny and Eissenstat, 2011);
- Transport fine roots (TR): non-mycorrhizal fine roots with secondary xylem which fulfill the role of water transport and starch and nutrient storage (McCormack et al., 2015);
- Absorptive mycorrhizal fine roots (MR): intensely branched fibrous fine roots, enlarging the plant absorptive surface by related symbionts (Agerer, 2001; McCormack et al., 2015).

In the experimental forest site, 7–10 sampling positions were randomly selected and marked within both beech and spruce groups. At each sampling position, one rootlet was entirely extracted from the topsoil, i.e. from the humus layer and the upper 10 cm of the mineral soil. Rootlets were subdivided into the three fine-root categories and dried to a constant weight, DW (g) (i.e., DW_FR_, DW_TR_, and DW_MR_) during the four sampling campaigns in 2003.

Dry masses were used to calculate the proportion of functional fine-root categories to individual measured parameters (e.g. RR, C, N, NSC) for an individual rootlet for each sampling campaign and tree species.

(1) $$X^{i}=\frac{X_{FR}\times DW_{FR}^{i}\;+\;X_{TR}\times DW_{TR}^{i}\;+\;X_{MR}\times DW_{MR}^{i}}{DW^{i}}$$

where X*^i^* is the parameter X calculated for the rootlet *i*; X_FR_, X_TR_ and X_MR_ are parameter levels each as derived from sub-samples of the fine-root categories FR, TR, and MR; DW*^i^*_FR_, DW*^i^*_TR_, and DW*^i^*_MR_ are dry masses (g) each of fine-root categories within a rootlet *i*, with DW*^i^* as total rootlet dry mass.

In some cases the transition from absorptive to transport fine-roots occurs gradually and may vary across species (McCormack et al., 2015). In a small pre-experiment, we determined fine root anatomy for our species to identify functional breakpoints. To this end, serial transverse sections (50 μm, from distal to proximal) were obtained in May and August on 7–10 fine-root segments of each tree species using a cryomicrotome (Frigocut, Reichert-Jung, Heidelberg, Germany). After staining with safranin and astrablue, the fine-root cross sections were mounted on glass slides and examined with a Trinocular Phase Contrast microscope (Leitz ARISTOPLAN, Leitz Meßtechnik GmbH, Germany) equipped with a digital color camera system (KAPPA model CF 20/4 DX; Kappa GmbH, Gleichen, Germany) and Kappa ImageBase 2.2 software. Anatomical study was, however, not in focus of the present investigation.

### Measurement of Fine-Root RR

The fine-root RR (nmol CO_2_ g^−1^ s^−1^) was measured by differential infrared gas analysis, IRGA (CIRAS-2, PP-Systems, UK) in combination with an open-chamber system (PLC Conifer, PP-Systems, UK). The window size of the conifer cuvette (70 x 50 mm) allowed RR measurement of fine-root sub-samples with a fresh weight ≤ 0.6 g. Corresponding sub-samples were taken from each fine-root category, cleaned by brushing off soil particles and dead root ramifications, and transferred into mesh bags each (60 x 40 mm, mesh size of 50 µm) to protect the analyzer from contamination. Empty bags were also run in preceding tests to ensure the absence of air contamination. Each mesh bag with its root sub-sample was immediately placed in the IRGA cuvette, operated with air humidity fixed to 90%, flow rate of 0.2 l min^−1^, and incoming CO_2_ concentration of 400 µl l^−1^. No CO_2_ contamination was detected in empty cuvettes with concentrations between 400 and 1,000 µl l^−1^ (also see Burton and Pregitzer, 2002). Cuvette temperature (T_c_) was set according to the measurement protocol (see below, ca. 7, 15, and 22°C). Root respiration was recorded upon stabilization, within 3–5 min after closing the cuvette. Since fine roots were not rinsed before measurements, microbial respiration included in the measurement RR, but was likely negligible (i.e. < 5% of RR, according to Burton and Pregitzer, 2003). Rates of microbial respiration per mass unit of soil debris are orders of magnitude lower than those of respiring, mass-related root tissue (Zak et al., 1999).

The relationship between fine-root development (see methodological details in Nikolova et al., 2006) and weather conditions (data not shown) permitted data pooling in four sampling campaigns in 2003, i.e., of April 15, April 24 and May 9 (spring), June 27 and July 4 (early summer), August 22 and 24 (late summer), and October 28, 30, and November 3 (autumn). Each time, RR was assessed for each fine-root category and tree species. Three T_c_ levels (7, 15, and 22°C) were applied to determine the temperature response of RR. To this end, extracted rootlets were covered with wet paper, transported to the lab in plastic bags and stored at approximately 12°C until measurement, within 3 h after sampling. Preliminary tests found RR remained stable within that time period. On each sampling date, three to six RR replicates were measured per root category, tree species and T_c_ level. A fresh root sub-sample was inserted at each temperature change. The RR response to T_c_ was examined for each fine-root category and sampling date by exponential regressions using *van’t Hoff* equation:

(2) $$RR=\;\rho \times e^{\theta \times Tc}$$

where *ρ* and *θ* are model coefficients, and RR is the RR of the respective fine-root category in beech or spruce. By means of Eq. 2, RR of each fine-root category was normalized to T_c_ = 10°C (i.e., RR^10^). RR^10^ was then calculated for each excavated rootlet and sampling date (Eq. 1). In addition, respiratory *Q_10_* were determined for each fine-root category:

(3) $$Q_{10}=\;e^{10\times \theta }$$

with *Q_10_* as the RR response to 10°C temperature change, and *θ* as model coefficient. Short-term *Q_10_* per each sampling date was distinguished from seasonal long-term *Q_10_* (Burton and Pregitzer, 2003). A *Q_10_* of about 1.0 reflects low temperature dependence of RR and thus low metabolic activity of roots (e.g., dormancy), whereas high *Q_10_* indicates highly active metabolism. RR upon determination for each fine-root category (nmol CO_2_ g^−1^ s^−1^) was scaled to the entire rootlet (Eq. 1). In September 2004, a RR reference measurement was taken at a temperature of 10°C in the three beech and spruce fine-root categories.

### Fine-Root Morphology

Four to six root samples per fine-root category of both beech and spruce were optically scanned after RR analysis (Scanner STD4800, Regent Instruments Inc., Canada). The scanner had an optical resolution of 300 dpi and pixel size of 0.085 mm which allowed measurements of root diameters > 0.18 mm (Biernacki and Lovett-Doust, 2002). Stored digital images where processed in batch mode using WinRHIZO^TM^ Pro analysis software (Regent Instruments Inc., Canada) to assess the SRA (cm^2^ g^−1^) and D (mm) of each fine-root category from each of the four sampling campaigns in 2003.

### Carbon and N Analysis

Scanned samples were then analyzed for C and N on 4–6 root sub-samples per fine-root category. Samples were dried at 65°C, milled and analyzed by combustion in an elemental analyzer (Leco, CHN1000, USA). Individual beech and spruce rootlet C and N concentrations were calculated according to Eq. 1. In September 2004, the fine-root samples used as RR reference were additionally analyzed for C and N content.

### Non-Structural Carbohydrate Analysis

In each sampling campaign, four to six rootlets per tree species were harvested and prepared for sugar and starch concentration analyses. Cut rootlets were covered with wet paper and transported in a plastic bag to the lab to avoid root damage and desiccation. Sub-samples from the three fine-root categories were carefully cleaned of soil particles and dead root ramifications by light brushing, frozen in liquid N, and stored at −80°C until analysis. The root samples were then ground manually in liquid N. Lyophilized, sugars (glucose, fructose, sucrose), and starch (hydrolyzed to glucose) were extracted according to Fleischmann et al. (2009) before measuring concentrations by HPLC. In spruce samples, pinitol was additionally identified (using expertise by M. Popp, University of Vienna, Austria). Total sugar concentration (TSC, mg g^−1^) was additionally calculated as the sum of all analyzed sugars, inclusive pinitol in case of spruce. Individual beech and spruce rootlet non-structural carbohydrates concentrations were calculated according to Eq. 1.

### Data Analysis

Seasonal effects (i.e., between the sampling campaigns) on fine-root parameters (i.e. SRA, D, non-structural carbohydrates, N and C concentrations, and C:N) were tested for each tree species by a factorial two-way ANOVA including fixed factors “category” and “sampling campaign”. The interaction term “category” × “sampling campaign” was also included to test whether the responses of different fine-root categories depend on the sampling timing. Model residuals were tested for normality (Shapiro-Wilk test) and variance homogeneity (Levene test). For multiple comparisons, subsequent Tukey-HSD post hoc tests (HSD.test function of the *agricolae* package in R; Mendiburu, 2020) were performed. The temperature effect on RR was assessed in each fine-root category by an exponential fit (Eq. 2), based on datasets measured at three temperature levels over four sampling campaigns in 2003. Effect of N on RR was analyzed with individual beech and spruce rootlets by standardized major axis regression models (*smatr* R package; Warton et al., 2012), using RR^10^ for standardization. Non-parametric *U*-test (Mann-Whitney *U* test) was used to test for significant differences between two sample units. Statistical evaluation was performed with SPSS (version 13.0, SPSS INC., Chicago, IL, USA) and R (version 3.4.4; R Development Core Team, 2018). In all analyses, differences at *p* < 0.05 were considered significant.

## Results

### Fine-Root Categories

**Figure 2:**
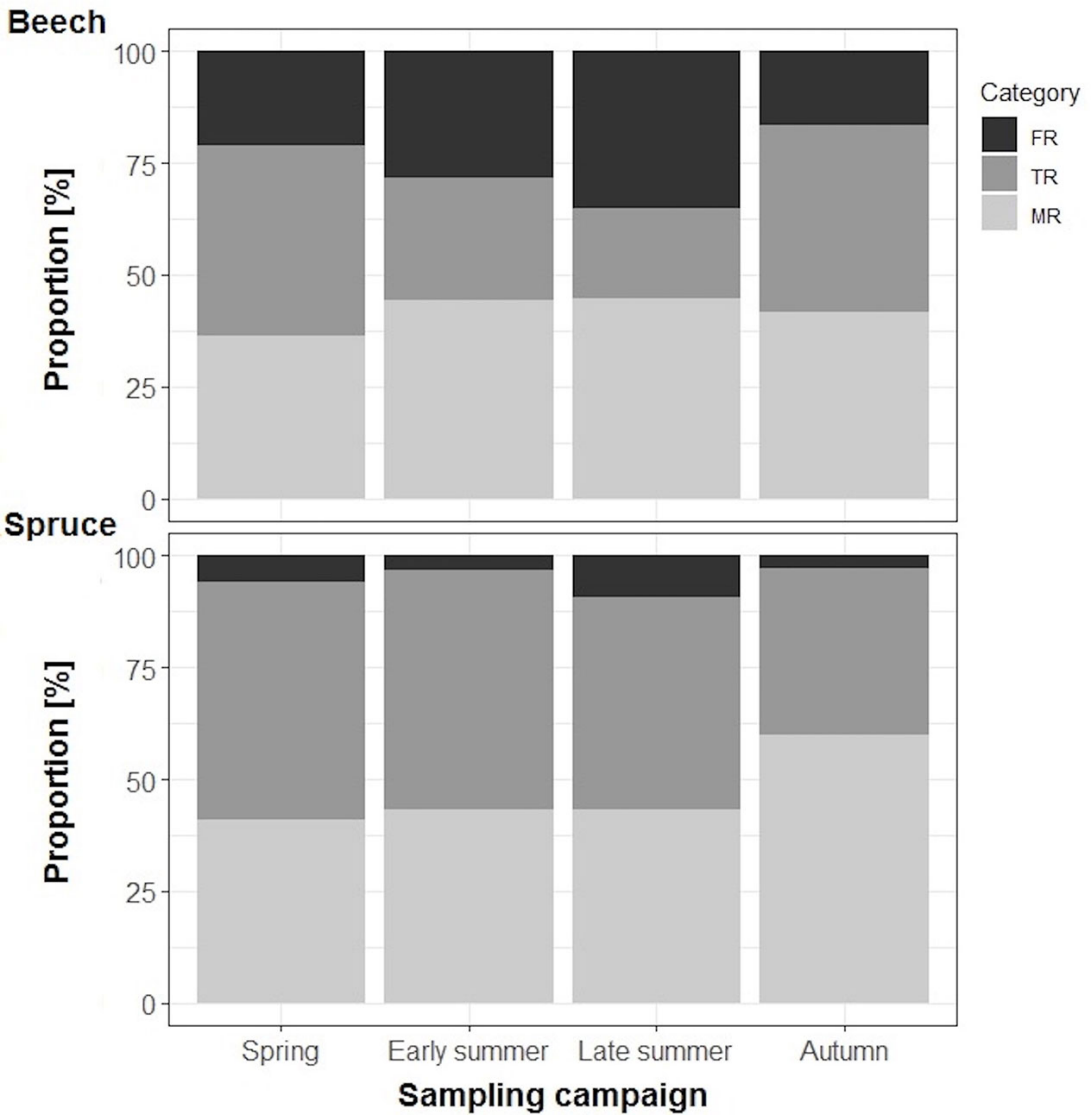
Average proportions (%) of three fine-root categories in the biomass of individual beech (n=7–8) and spruce (n=8–10) rootlets: FR = absorptive foraging roots with primary xylem, TR = transport roots with secondary xylem, MR = absorptive mycorrhizal fine roots.

Foraging roots (FR) constituted between 17–35% of the total beech rootlet biomass with a maximum of 35% in late summer and a minimum of 17% in autumn (**Figure 2**; **Table S1**). Transport fine roots (TR) contributed between 20-42% of the total rootlet biomass, with a minimum of 20% in late summer and a maximum of 42% in autumn, while mycorrhizal absorptive fine roots (MR) were consistently approximately 40% of the total rootlet biomass throughout the entire study period.

For spruce, FR contributed substantially less to the total rootlet biomass compared to beech and ranged between 10% in late summer and 3% in autumn (**Figure 2**; **Table S2**). Transport fine roots accounted for 37–53% of the total spruce fine-root biomass with a maximum of 53% in spring and a minimum of 37% in autumn. Absorptive mycorrhizal fine-roots contributed approximately 40% in spring through late summer and reached a maximum of 60% in autumn.

In spring, FR in both beech and spruce had whitish surface, but later, in early summer, spruce FR started to change color to yellow and brown (**Figure S1**). At the same time, beech FR remained whitish but appeared thinner and started to branch (**Figure S2**). In late summer of 2003, anatomical differences were apparent between beech and spruce FR: in beech, the living primary cortex was white, while in spruce, the primary cortex turned brown and shriveled (i.e., likely died), and the root surface suberized during drought (**Figure S1**).

### Fine-Root Morphology

In beech, SRA and D differed between fine-root categories depending on the season of the sampling campaign (**Table 2**). Beech FR SRA increased significantly from 167 cm^2^ g^−1^ in spring to a maximum of 533 cm^2^ g^−1^ in autumn. Beech SRA of the TR and MR categories were highest in early summer (78 and 520 cm^2^ g^−1^, respectively), but reached lowest levels in late summer (58 and 388 cm^2^ g^−1^); however this change was significant only for MR (**Table 1**). In beech, MR had generally the thinnest diameters (0.61–0.67 mm) and TR, had, in contrast, the largest diameters (1.40–1.62 mm). Interestingly, FR had diameters that were similar to TR at the beginning of the growing season (1.72 mm), but became thinner in early summer when their diameters were similar to those of MR (0.56 mm; **Table 1**).

**Table 1:** Specific fine-root area (SRA, cm^2^ g^−1^) and diameter (D, mm) in (A) beech and (B) spruce fine-root categories, assessed at four sampling campaigns during 2003.

Sampling campaign	Fine-root categories^*^					
	FR		TR		MR	
	SRA	D	SRA	D	SRA	D
**A) Beech**						
Spring	167 ± 15^d^	1.72 ± 0.22^a^	68 ± 6^e^	1.47 ± 0.08^bc^	440 ± 35^abc^	0.67 ± 0.06^d^
Early summer	482 ± 84^abc^	0.67 ± 0.05^d^	78 ± 4^e^	1.40 ± 0.08^c^	520 ± 64^ab^	0.61 ± 0.05^d^
Late summer	430 ± 172^bc^	0.69 ± 0.12^d^	58 ± 4^e^	1.62 ± 0.14^ab^	388 ± 103^c^	0.65 ± 0.08^d^
Autumn	553 ± 83^a^	0.56 ± 0.05^d^	65 ± 8^e^	1.51 ± 0.10^bc^	465 ± 73^abc^	0.65 ± 0.07^d^
**B) Spruce**						
Spring	145 ± 39^cd^	1.37 ± 0.28^cd^	109 ± 30^de^	1.54 ± 0.25^bc^	310 ± 27^ab^	0.84 ± 0.04^e^
Early summer	168 ± 36^c^	1.20 ± 0.22^d^	88 ± 17^e^	1.65 ± 0.13^b^	327 ± 30^a^	0.80 ± 0.04^e^
Late summer	140 ± 28^cd^	1.26 ± 0.40^d^	83 ± 11^e^	1.75 ± 0.18^ab^	299 ± 60^ab^	0.78 ± 0.07^e^
Autumn	143 ± 29^cd^	1.27 ± 0.40^d^	78 ± 10^e^	1.91 ± 0.20^a^	286 ± 21^b^	0.75 ± 0.04^e^

Means ± 1 standard deviation (n = 4–6). Differences between means sharing a letter (a = highest value) are not statistically significant (ANOVAs provided separately for beech (A) and spruce (B) with Tukey-HSD post hoc tests, p < 0.05).

^*^ FR, absorptive foraging fine roots; TR, transport fine roots; MR, absorptive mycorrhizal fine roots.

In spruce, SRA differed between fine-root categories and sampling campaigns with both factors independent from each other (**Table 2**). Spruce FR, in contrast to beech, had low seasonal variation of SRA (**Table 1**), reaching maximal levels in early summer (168 cm^2^ g^−1^), and minimal in late summer (140 cm^2^ g^−1^). TR had highest SRA in spring (109 cm^2^ g^−1^) and lowest in autumn (78 cm^2^ g^−1^). However, the changes in SRA of FR and TR were not significant relative to the other samplings. Spruce MR had the highest SRA among spruce roots with a maximum in SRA in early summer (327 cm^2^ g^−1^), and a minimum in autumn (286 cm^2^ g^−1^). Spruce D varied significantly between fine-root categories depending on the season of the sampling campaign (**Table 2**). Remarkably, no general seasonal adjustment of D was observed in spruce. In spruce, the diameter of FR roots was generally larger compared to beech FR, but in contrast to beech did not change with season (1.20–1.37 mm; **Table 1**). In spruce, TR had the largest diameter gradually increasing from 1.54 mm in spring to maximum of 1.91 mm in autumn. The MR category had the thinnest diameter (0.75–0.84 mm); however, the MR were coarser compared to the same category in beech.

**Table 2:** ANOVAs outcome for key fine-root parameters, assessed at four sampling campaigns during 2003 in fine-root categories of beech and spruce. Main factors are “category” and “sampling campaign” as well as their interaction term. Total number of samples for beech was 135, and for spruce 167.

Tree species		Beech		Spruce	
Response variable	*df*	MS	*F* value	MS	*F* value
**SRA**					
Category	2	2183794	315.9^***^	747037	662.9^***^
Sampling campaign	3	97257	14.1^***^	6423	5.7^***^
Category × sampling campaign	6	63994	9.3^***^	1305	0.3 ns
**D**					
Category	2	9.7	1031.0^***^	13.1	501.7^***^
Sampling campaign	3	0.6	67.5^***^	0.1	1.6 ns
Category × sampling campaign	6	0.6	66.4^***^	0.2	6.3 ^***^
**C concentration**					
Category	2	94.8	56.1^***^	9.1	54.3^***^
Sampling campaign	3	32.3	19.1^***^	7.1	42.5^***^
Category × sampling campaign	6	5.4	3.2^*^	0.8	4.35^**^
**N concentration**					
Category	2	5.9	273.3^***^	2.6	176.3^***^
Sampling campaign	3	0.7	33.4^***^	0.3	17.58^***^
Category × sampling campaign	6	0.6	26.5^***^	0.3	18.11^***^
**C/N**					
Category	2	5243	469.8^***^	936.3	118.5^***^
Sampling campaign	3	276.3	24.8^***^	94.7	12.0^***^
Category × sampling campaign	6	200.0	17.9^***^	91.7	11.6^***^
**Saccharose**					
Category	2	554.2	30.6^***^	228.1	7.8^**^
Sampling campaign	3	310.4	17.1^***^	1208	41.5^***^
Category × sampling campaign	6	154.1	8.5^***^	150.4	5.2^***^
**Glucose**					
Category	2	180.0	40.8^***^	62.9	12.1^***^
Sampling campaign	3	86.3	19.5^***^	417.8	80.2^***^
Category × sampling campaign	6	48.2	11.0^***^	187.6	36.0^***^
**Fructose**					
Category	2	284.1	44.9^***^	188.1	34.8^***^
Sampling campaign	3	104.9	16.6^***^	162.5	30.1^***^
Category × sampling campaign	6	23.7	3.8^**^	52.2	9.7^***^
**TSC**					
Category	2	1889	42.5^***^	3096	28.6^***^
Sampling campaign	3	1168	26.3^***^	5676	52.5^***^
Category × sampling campaign	6	73.4	1.7 ns	864.2	7.9^***^
**Starch**					
Category	2	7.3	34.2^***^	7.8	9.3^***^
Sampling campaign	3	1.4	6.4^**^	127.5	151.9^***^
Category × sampling campaign	6	3.7	17.1^***^	7.1	8.4^***^
**Pinitol**					
Category	2	nd	nd	884.9	105.2^***^
Sampling campaign	3	nd	nd	182.1	21.6^***^
Category × sampling campaign	6	nd	nd	51.0	6.1^***^

For multiple comparisons, Tukey-HSD post hoc tests (p < 0.05) were performed.

df, degrees of freedom; MS, mean square. F value with significance levels ***p < 0.001; **p < 0.01; *p ≤ 0.05; ns p > 0.05; nd = not defined.

### Fine-Root Respiration

**Figure 3:**
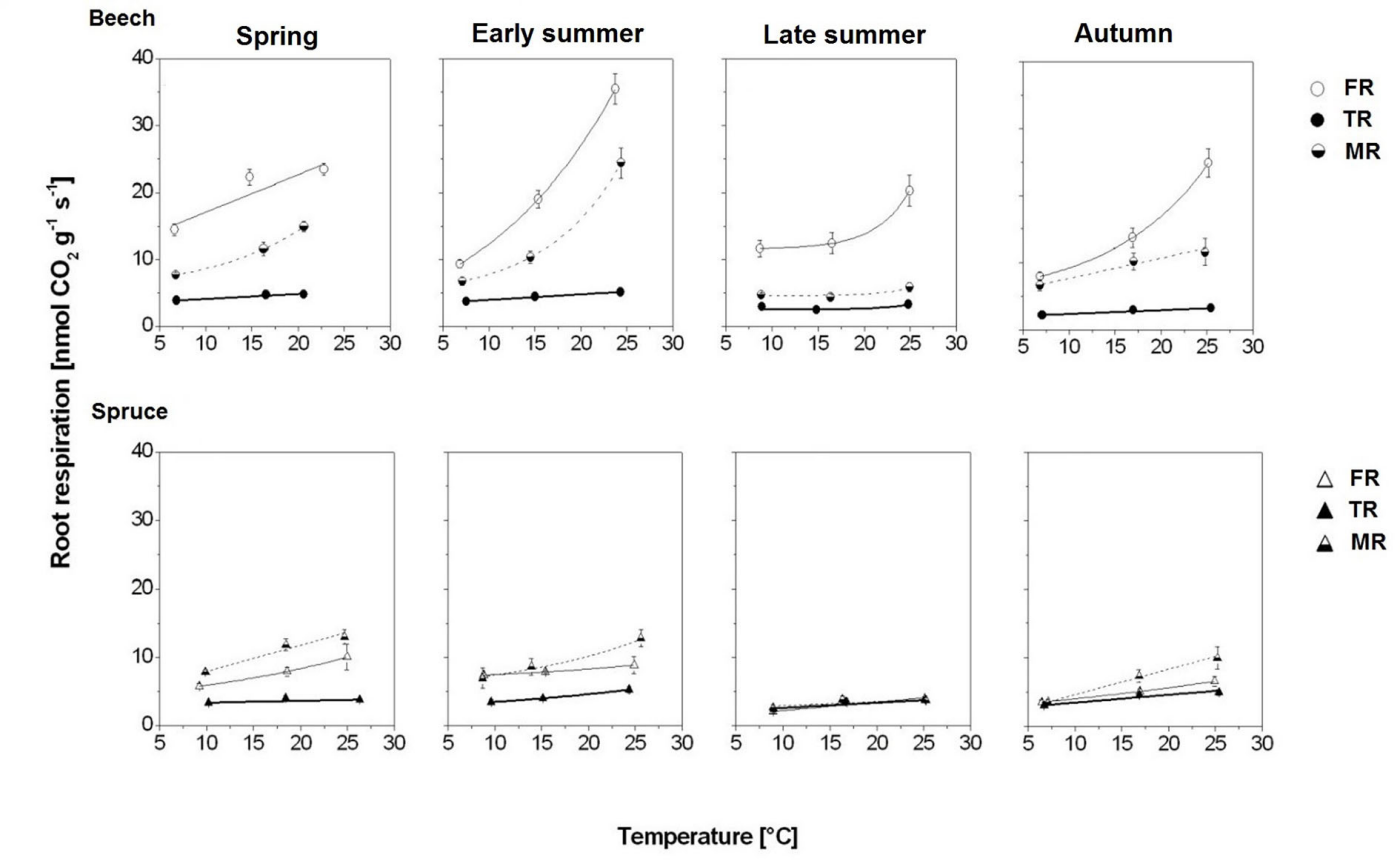
Seasonal response of root respiration rate (RR) to temperature in beech and spruce per fine-root category, given as exponential response functions of absorptive foraging fine roots FR (solid thin line), transport fine roots TR (solid bold line), and absorptive mycorrhizal fine roots MR (dashed line); means ± 1 standard error (n = 4–6). Model statistics are provided in **Table 3**.

In beech, FR had the highest RR^10^ and temperature sensitivity (**Figure 3**; **Table 3**): RR^10^ ranged between 17.06 nmol CO_2_ g^−1^ s^−1^ in spring and 9.46 in autumn, and *Q_10_* was highest in early summer (i.e., 2.20) but lowest in spring and late summer (i.e., around 1.4). In contrast, TR had the lowest RR^10^ (4.14–2.40 nmol CO_2_ g^−1^ s^−1^) and temperature sensitivity: *Q_10_* even dropped to 1.09 in the extremely dry month of August (i.e., late summer). On average, MR had RR^10^ levels of 4.5 CO_2_ g^−1^ s^−1^ (late summer) to 8.9 nmol CO_2_ g^−1^ s^−1^ (spring), with the lowest *Q_10_* of 1.16 in late summer.

**Table 3:** Root respiration rate (RR) in response to temperature per fine-root category of beech (A) and spruce (B) at four sampling campaigns during 2003.

Samplingcampaign	Fine-root category^*)^	Model parameters		*Q_10_*	*R^2^*	RR^10^(nmol CO_2_g^−1^s^−1^)
		*ρ* (nmol CO_2_g^−1^s^−1^)	*θ* (°C)			
**A) Beech**						
Spring	FR TR MR	12.66 3.51 5.58	0.029 0.047 0.017	1.35 1.18 1.60	0.83 0.96 0.99	17.06 4.14 8.90
Early summer	FR TR MR	5.53 3.33 3.79	0.079 0.018 0.075	2.20 1.20 2.12	0.99 0.97 0.99	12.16 4.04 8.02
Late summer	FR TR MR	8.06 2.54 3.90	0.035 0.009 0.015	1.41 1.09 1.16	0.85 0.25 0.60	11.38 2.77 4.50
Autumn	FR TR MR	5.11 1.94 5.55	0.062 0.022 0.031	1.85 1.24 1.37	0.99 0.93 0.95	9.46 2.40 7.59
**B) Spruce**						
Spring	FR TR MR	4.18 3.28 5.75	0.035 0.007 0.035	1.42 1.10 1.42	0.99 0.43 0.94	5.93 3.52 8.15
Early summer	FR TR MR	6.79 2.61 4.90	0.010 0.029 0.039	1.11 1.37 1.47	0.68 0.99 0.99	7.53 3.49 7.19
Late summer	FR TR MR	1.52 2.03 2.43	0.041 0.026 0.019	1.50 1.30 1.20	0.88 0.82 0.61	2.28 2.64 2.95
Autumn	FR TR MR	2.85 2.62 2.47	0.04 0.027 0.058	1.40 1.30 1.80	0.99 0.89 0.96	4.00 3.44 4.41

Exponential fit (RR = ρe^θTc^) describes the relationship between RR and T_c_ (Eq. 2), with ρ and θ being model parameters; Q_10_ calculated as Q_10_ = e^10θ^ (Eq. 3); R^2^ as coefficient of determination (n = 4–6); RR^10^ at standard T of 10°C.

^*^FR, absorptive foraging fine roots; TR, transport fine roots; MR, absorptive mycorrhizal fine roots.

Compared to beech, all fine-root categories of spruce had lower respiration levels and less variation in RR^10^ throughout the entire study period. Spruce FR had 2–3 times lower RR^10^ but comparable *Q_10_* except early summer when *Q_10_* in spruce FR was extremely low (i.e., 1.11; **Figure 3**; **Table 3**). Spruce TR had the lowest RR^10^ and *Q_10_*, ranging between 2.6 and 3.5 nmol CO_2_ g^−1^ s^−1^, and 1.09 and 1.37, respectively. The highest RR^10^ and temperature sensitivity were found in spruce MR (**Figure 3**; **Table 3**): RR^10^ maximum in spring (8.15 nmol CO_2_ g^−1^ s^−1^), and *Q_10_* maximum of 1.80 after precipitation returned in autumn (**Figure 1**).

Rootlet RR at mean daily soil temperature (RR^T(0)^) and at a standard temperature of 10°C (RR^10^) were higher in beech than spruce over the entire study period (**Figure S3**). In late summer, rootlet RR^T(0)^ decreased significantly in both tree species despite higher soil temperature (T_s_). Beech rootlets had the highest RR^10^ in spring (9.90 nmol CO_2_ g^−1^ s^−1^). For the rest of the season, rootlet RR were similar in both species with the seasonal RR^10^ minimum occurring in late summer. In autumn 2004, rootlet RR^10^ was, in both tree species, 30–50% higher compared to autumn 2003, with higher increases in spruce.

### Carbon and N Status

Carbon and N concentrations varied among transport versus absorptive fine-root categories and sampling periods (**Table 2**). In beech, FR and MR had the lowest C concentration in spring (400 mg g^−1^ to 420 mg g^−1^) that increased over the course of the season up to nearly 430–450 mg g^−1^ (**Table 4**). Transport root C concentration was generally higher than in the other two categories, and similarly increased across the entire sampling period (i.e., from 467 mg g^−1^ to 476 mg g^−1^). During late summer, FR reached maximal N concentrations among all categories (27.2 mg g^−1^); however, these patterns shifted by autumn where FR and MR had similar N concentrations (nearly 19 mg g^−1^). This similar N level was the result in a drop in N in the FR roots at the end of vegetation period. Across the entire study period, TR had the lowest N concentration among all categories, ranging from 6.8 mg g^−1^ in early summer to 10.1 mg g^−1^ in autumn. The C:N ratio was lowest in FR (a minimum of 16.6 mg g^−1^ in late summer and a maximum of 29.5 mg g^−1^ in spring), and highest in TR (a minimum of 47.2 mg g^−1^ in autumn a maximum of 69.5 mg g^−1^ in early summer), with lowest seasonal variation in MR category (23–32 mg g^−1^). When calculated per rootlet, C:N of beech rootlets were the lowest in late summer, a result of the high proportion of high N containing FR roots that were present at this time (**Table 4**, **Figure S4**).

**Table 4:** Concentration of carbon (C, mg g^−1^) and nitrogen (N, mg g^−1^), and the C:N ratio in the fine-root categories of (A) beech and (B) spruce at four sampling campaigns during 2003.

Sampling campaign	Fine-root categories^*^									Rootlet		
	FR			TR			MR					
	C	N	C:N	C	N	C:N	C	N	C:N	C	N	C:N
**A) Beech**												
Spring	400 ± 9.3^e^	13.8 ± 2.0^d^	29.5 ± 3.7^cd^	467 ± 3.3^ab^	9.6 ± 0.7^e^	49.0 ± 3.3^b^	420 ± 1.2^de^	16.4 ± 1.0^cd^	25.6 ± 1.3^cde^	*442 ± 2.9^a^*	*15.8 ± 1.5^a^*	*28.3 ± 2.8^a^*
Early summer	444 ± 9.4^bcd^	22.0 ± 2.0^b^	20.3 ± 1.9^ef^	473 ± 2.5^ab^	6.8 ± 0.6^e^	69.5 ± 6.4^a^	458 ± 2.2^abc^	14.2 ± 1.1^d^	32.5 ± 2.9^c^	*459 ± 2.2^b^*	*14.5 ± 1.2^a^*	*31.8 ± 2.6^a^*
Late summer	449 ± 6.0^abc^	27.2 ± 1.4^a^	16.6 ± 0.9^f^	473 ± 1.3^ab^	9.4 ± 1.1^e^	50.9 ± 5.9^b^	458 ± 4.3^abc^	18.4 ± 1.2^bc^	24.8 ± 1.6^cde^	*458 ± 2.7^b^*	*19.4 ± 1.8^b^*	*23.8 ± 2.3^b^*
Autumn	431 ± 3.1^cd^	19.2 ± 0.9^bc^	22.5± 2.2^def^	476 ± 8.4^a^	10.1 ± 0.5^e^	47.2 ± 3.0^b^	447 ± 2.2^bcd^	19.7 ± 2.8^b^	23.0 ± 2.7^def^	*446 ± 2.4^a^*	*15.7 ± 0.6^a^*	*29.2 ± 1.1^a^*
**B) Spruce**												
Spring	448 ± 5.7^d^	12.7 ± 0.7^efg^	35.2 ± 1.6^bc^	463 ± 2.1^bc^	12.0 ± 1.0^fg^	38.9 ± 3.2^ab^	463 ± 3.2^bc^	22.2 ± 0.7^a^	20.9 ± 1.6^f^	*462 ± 0.5^a^*	*17.6 ± 0.6^a^*	*26.3 ± 0.9^a^*
Early summer	450 ± 3.1b^d^	16.5 ± 0.9^cd^	27.4 ± 1.9^de^	462 ± 6.1^bc^	10.4 ± 0.8^g^	44.9 ± 4.4^a^	466 ± 3.3^bc^	14.7 ± 1.0^de^	31.9 ± 2.2^cd^	*464 ± 0.8^b^*	*13.0 ± 0.4^b^*	*35.8 ± 1.0^b^*
Late summer	460 ± 4.0^c^	14.0 ± 0.7^def^	32.9 ± 1.6^bcd^	462 ± 1.9^bc^	11.8 ± 1.4^fg^	39.6 ± 5.2^ab^	467 ± 0.5^bc^	19.2 ± 0.5^bc^	24.3 ± 0.7^ef^	*464 ± 0.5^b^*	*15.5 ± 0.7^c^*	*30.1 ± 1.3^c^*
Autumn	467 ± 2.7^bc^	16.6 ± 2.1^cd^	28.7 ± 4.3^de^	470 ± 6.6^a^	14.5 ± 0.9^def^	32.7 ± 2.1^cd^	482 ± 4.0^a^	20.5 ± 1.2^a^	23.6 ± 1.4^ef^	*474 ± 0.8^c^*	*16.8 ± 0.4^a^*	*28.2 ± 0.7^ca^*

Means ± 1 standard deviation (n = 4–6). Differences between means sharing a letter (a = highest value) are not statistically significant (ANOVAs provided separately for beech (A) and spruce (B) with Tukey-HSD post hoc tests, p < 0.05). Values representative for entire rootlets were calculated according to Eq.1 (see also **Figure S4**).

^*^FR, absorptive foraging fine roots; TR, transport fine roots; MR, absorptive mycorrhizal fine roots.

In spruce, C was more stable across all fine-root categories and months (**Table 4**) with lowest levels in FR during spring (448 mg g^−1^) and highest in MR during autumn (482 mg g^−1^). In contrast to beech, TR was not the C-richest category in spruce, but was similar to MR (C differences between both categories were not significant). Absorptive mycorrhizal roots had generally the highest N levels, with the exception of early summer when N decreased substantially (14.7 mg g^−1^) to the N-levels of FR (i.e., nearly 16 mg g^−1^). Similar to beech, TR had the lowest N concentration among the fine-root categories, especially in early and late summer (10–12 mg g^−1^). In contrast to beech, spruce rootlets had the highest C:N in early summer, which was due to the reduced N at the beginning of the drought period (**Table 3**; **Figure S4**). In autumn 2004, N content in spruce rootlets was nearly 30% higher compared to autumn 2003, indicating N limitation in spruce at the end of the growing season in 2003 (**Figure S4**).

### Relationship Between RR and N Concentration

**Figure 4:**
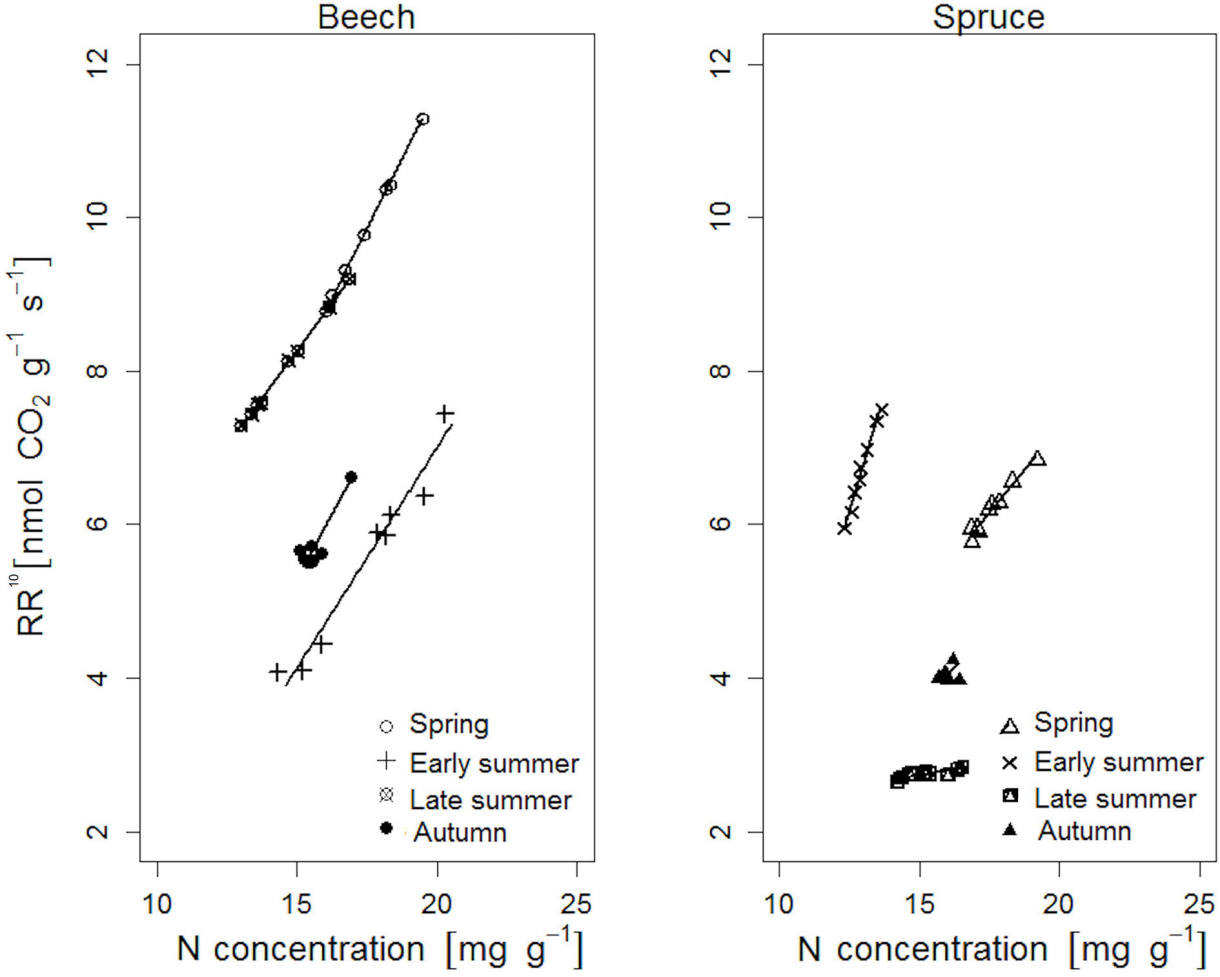
Relationship between root respiration rate at a standard temperature of 10°C (RR^10^) and nitrogen concentration of individual beech and spruce rootlets. Lines represent a linear fit (regression statistics are provided in **Table 5**).

In both tree species, RR^10^ was positively correlated to the corresponding N concentration of the rootlets (**Figure 4**). In beech, RR^10^ peaked in response to N in spring (*β* = 7.27, *p* < 0.001), compared to early spring in spruce (*β* = 12.18, *p* < 0.001; **Table 5**). Beech RR^10^ responded positively to N across all sampling dates (coefficient *β* between 4.97 and 7.27), while spruce responded weakly (late summer: *β* = 0.61, *p* = 0.001), or not at all (autumn: *β* = 3.65, *p* = 0.478; **Table 5**).

**Table 5:** Linear fit (RR^10^ = *α* + *β*N) between root respiration rate (RR) at a standard temperature of 10°C (RR^10^; nmol CO_2_ g^−1^ s^−1^) and nitrogen concentration (N, mg g^−1^) in individual rootlets of (A) beech and (B) spruce at four sampling campaigns during 2003.

Sampling campaign 2003	Intercept, *α*(upper/lower limit)	Slope, *β*(upper/lower limit)	n	*R^2^*	*p*
**A) Beech**					
Spring	−2.86 (− 3.30/−2.43)	7.27 (7.03/7.52)	7	0.99	<0.0001
Early summer	0.81 (0.56/1.03)	4.97 (4.82/5.13)	8	0.99	<0.0001
Late summer	−4.48 (−6.46/−2.50)	5.63 (4.73/6.98)	8	0.96	<0.0001
Autumn	−4.71 (−9.54/0.12)	6.67 (4.27/10.44)	8	0.78	0.003
**B) Spruce**					
Spring	−1.64 (−3.16/−0.12)	4.46 (3.68/5.40)	9	0.95	<0.0001
Early summer	−9.05 (−10.87/−7.23)	12.18 (10.85/13.67)	8	0.99	<0.0001
Late summer	1.81 (1.42/2.20)	0.61 (0.40/0.92)	10	0.74	0.001
Autumn	−1.79 (−6.81/3.21)	3.65 (1.67/7.95)	9	0.07	0.478

Here, α (intercept) and β (slope) are regression coefficients, n is the number of the analyzed individual rootlets, R^2^ represents the measure of determination, p gives the level of significance of the regression equations (standardized major axis regression models). Upper and lower limits of α and β are shown in brackets.

### Non-Structural Carbohydrates

All studied non-structural carbohydrates varied among fine-root categories and sampling periods (**Table 2**). Interestingly, the seasonal responses of NSC in beech were similar among the studied fine-root categories, i.e. were independent on the sampling timing. During spring and early summer 2003, beech FR had the lowest TSC concentration 38 mg g^−1^ (**Table 6**; **Figure S5**), which increased by 40–60% during late summer the second half of the growing season as a result of an increase in sucrose and fructose (in late summer), and glucose concentrations (in autumn). Transport fine-roots of beech had lowest TSC also in spring (35.2 mg g^−1^), but peaked in the second half of the growing season also due to enhanced fructose and glucose concentration. Absorptive mycorrhizal fine-roots of beech had low TSC during spring and early summer (approximately 20 mg g^−1^), but levels doubled during late summer and autumn similar to FR. The three fine-root categories of beech showed however different patterns of starch allocation during the study period (**Table 6**): highest concentration was detected in FR in spring (3.26 mg g^−1^) and steadily decreased through the rest of the growing season (**Figure S5**). In TR starch was extremely low in spring and then completely exhausted in early summer. Interestingly, the starch levels in TR recovered in late summer and in autumn; this seasonal change was however not significant. Absorptive mycorrhizal roots had oscillating starch levels, with minimal levels in late summer, but accumulated starch in autumn (1.86 mg g^−1^).

**Table 6:** Non-structural carbohydrate concentrations in fine-root categories of (A) beech and (B) spruce in 2003.

A) Beech								
Fine-root category	Sampling campaign	Sucrose (mg g^−1^)	Glucose (mg g^−1^)		Fructose (mg g^−1^)	TSC (mg g^−1^)	Starch (mg g^−1^)	
FR	Spring	24.4 ± 3.3^bc^	7.3 ± 0.9^cde^		8.2 ± 1.7^cde^	38.2 ± 3.9^bc^	3.26 ± 1.05^a^	
	Early summer	20.7 ± 5.1^bcd^	9.5 ± 1.8^bc^		8.5 ± 1.6^cde^	38.8 ± 8.2^bc^	1.56 ± 0.38^bc^	
	Late summer	38.6 ± 4.5^a^	8.3 ± 2.7^bcd^		12.1 ± 3.4^bcd^	60.1 ± 6.0^a^	1.42 ± 0.51^bc^	
	Autumn	29.1 ± 7.2^b^	18.9 ± 3.3^a^		8.4 ± 1.9^cde^	52.6 ± 10.3^ab^	0.56 ± 0.08^cd^	
TR	Spring	23.8 ± 1.6^bc^	3.8 ± 0.5^de^		7.4 ± 1.2^de^	35.2 ± 2.7^c^	0.55 ± 0.41^cd^	
	Early summer	21.9 ± 1.1^bc^	8.7 ± 0.5^bcd^		13.6 ± 0.7^abc^	44.2 ± 1.8^bc^	0^d^	
	Late summer	19.4 ± 6.2^cd^	12.0 ± 3.2^b^		17.9 ± 5.2^a^	49.5 ± 9.9^abc^	0.84 ± 0.29^bcd^	
	Autumn	20.5 ± 2.7^bcd^	9.8 ± 3.8^bc^		17.5 ± 6.3^ab^	48.1 ± 8.4^abc^	0.70 ± 0.35^cd^	
MR	Spring	12.1 ± 3.6^de^	3.7 ± 1.4^e^		4.4 ± 1.3^e^	20.1 ± 5.9^d^	0.20 ± 0.04^d^	
	Early summer	8.8 ± 1.5^e^	4.4 ± 0.8^de^		4.9 ± 1.0^e^	18.1 ± 2.8^d^	0.17 ± 0.02^d^	
	Late summer	24.9 ± 5.1^bc^	5.3 ± 1.1^cde^		8.2 ± 1.9^cde^	38.7 ± 6.7^bc^	0^d^	
	Autumn	24.1 ± 1.9^bc^	4.5 ± 0.7^de^		7.9 ± 4.1^cde^	37.0 ± 3.1^c^	1.86 ± 0.20^b^	
*Rootlet*	Spring	*19.6 ± 0.8^c^*	*4.5 ± 0.3^c^*		*6.4 ± 0.2^c^*	*30.6 ± 1.3^c^*	*1.02 ± 0.27^ac^*	
	Early summer	*15.8 ± 0.8^d^*	*7.0 ± 0.4^b^*		*8.3 ± 0.4^b^*	*31.1 ± 1.4^c^*	*0.94 ± 0.10^ac^*	
	Late summer	*28.3 ± 1.7^a^*	*7.9 ± 0.7^b^*		*10.6 ± 1.4^a^*	*46.9 ± 1.5^a^*	*0.82 ± 1.14^b^*	
	Autumn	*23.3 ± 0.5^b^*	*9.1 ± 0.7^a^*		*12.1 ± 0.7^a^*	*44.5 ± 1.0^b^*	*1.18 ± 0.09^a^*	
**B) Spruce**								
		**Sucrose (mg g^−1^)**	**Glucose (mg g^−1^)**		**Fructose (mg g^−1^)**	**Pinitol (mg g^−1^)**	**TSC (mg g^−1^)**	**Starch (mg g^−1^)**
FR	Spring	51.9 ± 11.5^a^	7.4 ± 4.5^cde^		13.6 ± 3.8^bc^	15.1 ± 4.0^b^	88.1 ± 22.8^b^	5.67 ± 1.75^b^
	Early summer	41.0 ± 7.9^abc^	32.8 ± 5.1^a^		20.8 ± 2.8^a^	29.2 ± 1.1^a^	123.9 ± 14.0^a^	1.40 ± 0.22^c^
	Late summer	40.7 ± 2.8^abc^	9.0 ± 3.8^cd^		8.5 ± 1.9^cde^	24.8 ± 7.4^a^	83.0 ± 13.1^bc^	0.58 ± 0.13^c^
	Autumn	20.6 ± 2.6^e^	1.2 ± 0.6^f^		3.5 ± 0.9^e^	13.0 ± 3.1^bc^	38.4 ± 2.7^fg^	1.27 ± 0.50^c^
TR	Spring	30.1 ± 2.9^cde^	5.9 ± 1.3^def^		12.7 ± 1.0^bc^	6.8 ± 1.3^cd^	55.5 ± 5.0^defg^	10.85 ± 1.50^a^
	Early summer	43.5 ± 5.7^ab^	14.7 ± 1.8^b^		16.1 ± 3.5^ab^	9.8 ± 1.9^bcd^	84.1 ± 10.7^bc^	1.88 ± 1.50^c^
	Late summer	36.7 ± 1.4^bc^	12.4 ± 1.4^bc^		17.0 ± 2.3^ab^	12.1 ± 2.6^bc^	78.3 ± 3.0^bcd^	0.90 ± 0.54^c^
	Autumn	21.4 ± 7.3^de^	5.4 ± 1.7^def^		12.0 ± 4.1^bc^	5.6 ± 1.7^d^	44.4 ± 13.5^efg^	2.10 ± 1.38^c^
MR	Spring	37.1 ± 3.3^bc^	9.6 ± 0.9^bcd^		9.3 ± 0.9^cd^	5.7 ± 0.4^d^	61.8 ± 4.6^cde^	6.20 ± 0.74^b^
	Early summer	35.3 ± 2.6^bc^	7.4 ± 0.6^cde^		10.0 ± 0.5^cd^	6.5 ± 0.9^cd^	59.1 ± 3.5^cdef^	1.40 ± 0.83 ^c^
	Late summer	33.1 ± 3.8^bcd^	7.4 ± 1.6^cde^		9.0 ± 1.1^cd^	7.3 ± 2.4^cd^	56.9 ± 5.8^def^	0.96 ± 0.06^c^
	Autumn	21.1 ± 3.3^e^	3.2 ± 0.6^ef^		4.6 ± 0.5^de^	4.0 ± 1.0^d^	32.8 ± 4.7^g^	0.76 ± 1.17^c^
*Rootlet*	Spring	*35.3 ± 0.7*^b^	*8.0 ± 0.2*^c^		*10.9 ± 0.2*^b^	*6.7 ± 0.3*^b^	*60.9 ± 1.0^c^*	*7.99 ± 0.31^a^*
	Early summer	*38.9 ± 0.5*^a^	*11.1 ± 1.2*^a^		*10.3 ± 0.3*^b^	*8.5 ± 0.8*^a^	*68.8 ± 3.3^a^*	*1.59 ± 0.04^b^*
	Late summer	*35.3 ± 0.4*^b^	*9.7 ± 0.5*^b^		*12.5 ± 0.8*^a^	*6.5 ± 1.2*^bc^	*64.1 ± 1.3^b^*	*0.90 ± 0.02^c^*
	Autumn	*21.2 ± 0.1*^c^	*4.4 ± 0.3*^d^		*8.9 ± 0.7*^c^	*5.4 ± 0.3*^c^	*39.8 ± 0.9^d^*	*1.56 ± 0.11^b^*

**Figure 5:**
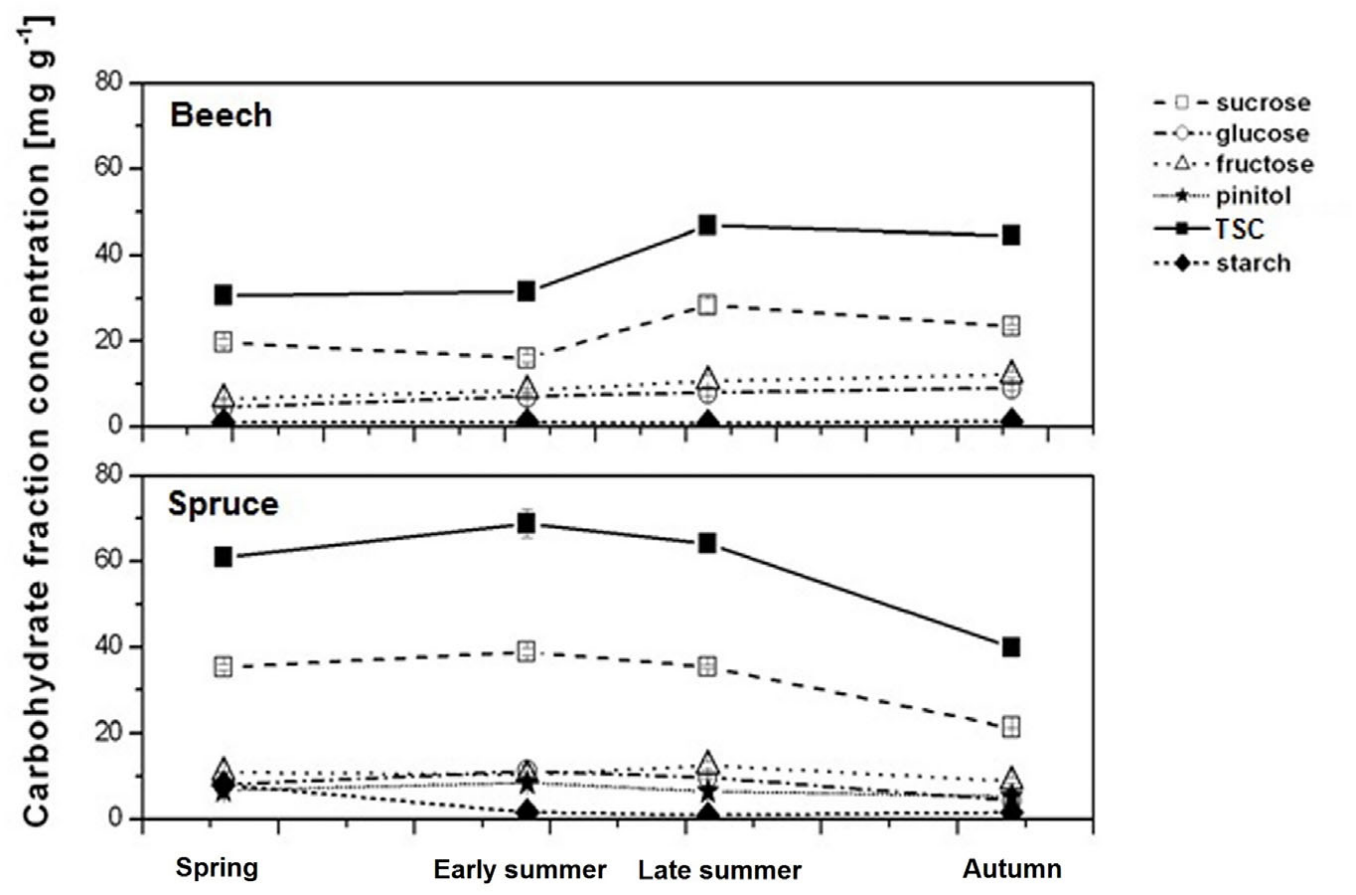
Concentrations of the carbohydrate fractions (sucrose, glucose, fructose, pinitol) and the total sugar concentration TSC (i.e. integral of sucrose, glucose, fructose, and, in case of spruce, pinitol) together with starch concentration in individual rootlets of beech and spruce during 2003 (means ± 95% confidence interval, n = 7–10).

Means ± 1 standard deviation (n = 4–6). Means with the same letter (a = highest value) are not statistically significant (ANOVAs provided separately for beech (A) and spruce (B) with Tukey-HSD post hoc tests, p < 0.05). Values representative for entire rootlets were calculated according to Eq.1 (see also **Figure 5**).

Fine-root category: FR, absorptive foraging fine roots; TR, transport fine roots; MR, absorptive mycorrhizal fine roots, TSC, total sugar concentration (sucrose, glucose, fructose, and pinitol in case of spruce).

Spruce fine-root categories had more variable seasonal patterns in NSC fractions compared to beech (**Table 2** and **Table 6**). Foraging non-mycorrhizal roots had highest TSC in early summer (123.9 mg g^−1^), and lowest in autumn (38.4 mg g^−1^), with glucose, fructose and pinitol influencing the seasonal variation (**Figure S5**). Total sugar concentration in TR of spruce also had the highest levels in early summer (84.1 mg g^−1^), but minimum in autumn (44.4 mg g^−1^), although the seasonal variation, driven mainly by sucrose, was less pronounced than in FR. In MR, TSC had highest levels in spring (61.8 mg g^−1^), and lowest in autumn (32.8 mg g^−1^) due to a simultaneous decrease in the concentration of all analyzed sugars. Starch concentrations in fine-root categories of spruce were highly variable at the beginning of the study (6–11 mg g^−1^ in spring), but then decreased five-fold and leveled out at similar concentrations for the remainder of the study (1–2 mg g^−1^; **Table 6**). Interestingly, TR had in spring starch levels of 10.85 mg g^−1^ that were approximately two-fold greater than FR or MR roots but, then decreased five-to-ten-fold to levels typical for the other two categories.

At the beginning of the growing season, beech rootlets had two times lower TSC relative to spruce (**Figure 5**). During late summer and autumn, however, TSC of beech rootlets increased by 50%. In spruce, TSC was only slightly enhanced in early summer, remained stable into late summer, but decreased in autumn. In both species, the seasonal dynamics of TSC was mainly driven by sucrose which contributed 55–65% to TSC. In general, the higher sucrose levels during spring and summer period as well as the permanent presence of pinitol resulted in the higher TSC in spruce relative to beech fine-roots. Rootlet starch concentration in beech did not show pronounced seasonal dynamics (**Table 6**, **Figure 5**). Despite higher levels of starch in spruce during spring, by the end of the growing season starch levels were similar in both beech and spruce (i.e., about 1.0–1.5 mg g^−1^) (**Table 6**; **Figure 5**).

## Discussion

**Figure 6:**
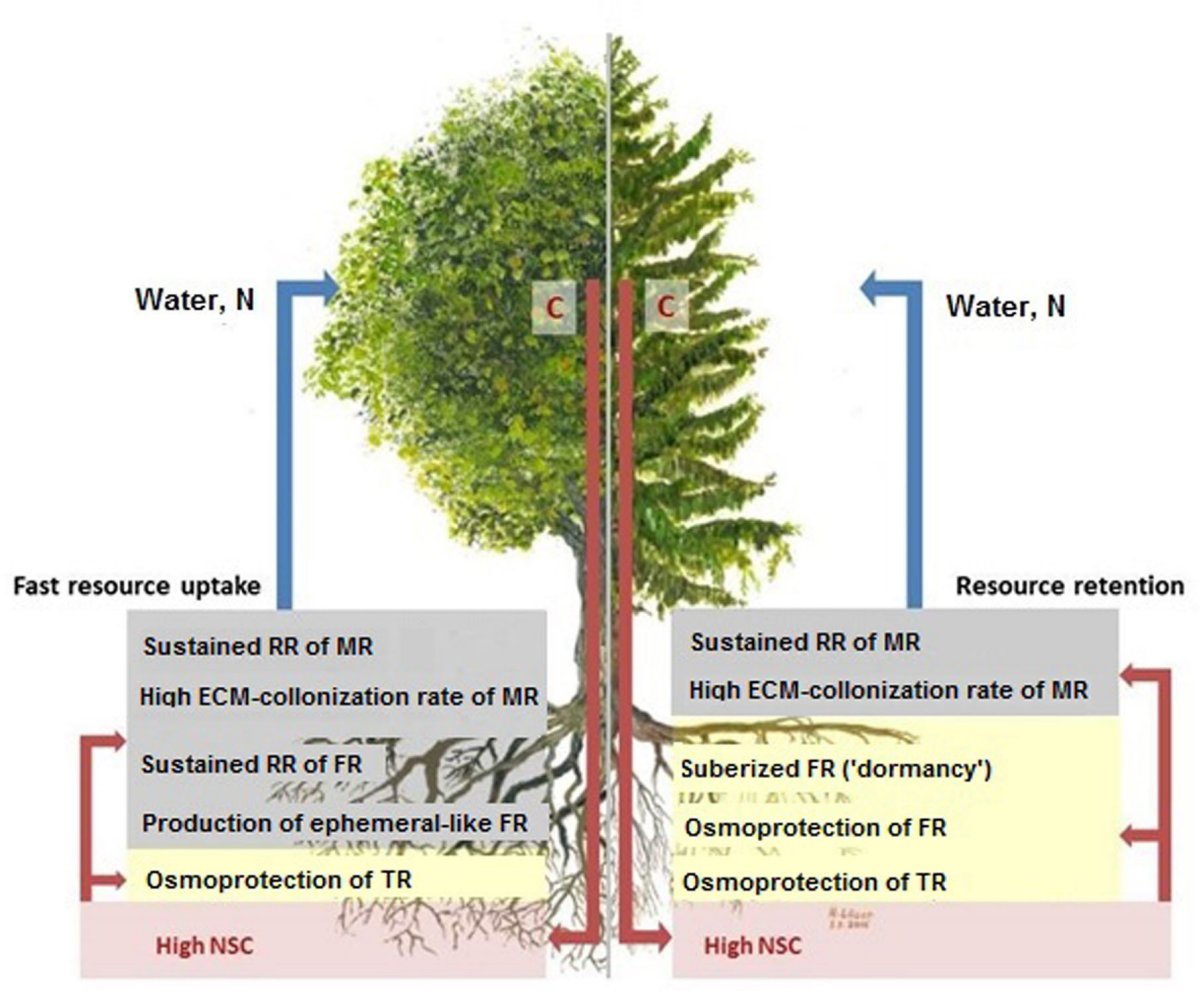
Scheme of the two different ecological strategies of beech (left) and spruce (right) fine-roots under seasonal drought. Red arrows show the transport of C (assimilates, NSC) from leaves into root structures and functions, blue arrows show the returns of soil resources (e.g., water, N) from roots to leaves. The fine-root traits associated with “fast” ecological strategy are indicated in grey, those associated with “slow” ecological strategy are shown in yellow. The NSCs (red lettering) are crucial in linking and controlling above and belowground processes. ECM-colonization data according to Nikolova, 2007. Abbreviations see text.

Our study presents a novel look at beech and spruce fine-root ecological strategies (cf. Weemstra et al., 2017) by directly comparing key morphological and physiological traits of three functionally defined fine-root categories. We hypothesized that the fine-roots of both species would adjust to seasonal drought differently with beech following a “fast”, and spruce a “slow” ecological strategy. However, we determined that each tree species employs diverse ecological strategies dependent on the functional fine-root categories investigated (**Figure 6**).

### Morphological Traits

The non-mycorrhizal absorptive roots adjusted in a species-specific manner supporting fine-root ecological strategy hypothesis. In beech, SRA of FR had the highest seasonal variation among all categories that more than doubled from spring through autumn. Enhanced SRA of FR was accompanied by a distinct reduction in mean diameter to the levels typical of mycorrhizal MR. Such a diameter decline may be associated with optimized uptake of scarce resources (Zobel et al., 2007; Ma et al., 2018). During drought, thin ephemeral roots are “shed” by species which are capable of root regrowth upon soil rewetting (Meier and Leuschner, 2008). The ability to “shed” roots and to initiate new root growth when available resources are present, represents an effective adaptation to drought, as long as the trees can afford the C demand (Brunner et al., 2015). This “shed-regrowth” mechanism as detected here in FR allows beech to regain fine-root biomass after drought (Leuschner et al., 2001). While substantial fine-root growth after drought comes at a C cost, the ability to grow new absorptive fine-roots with high SRA is essential for continued water uptake, demonstrating a “fast” ecological strategy for FR (**Figure 6**).

In contrast, spruce FR showed no seasonal variation of SRA. Instead, spruce FR became pigmented from white to brown during the dry midsummer (Blaschke et al., 2006; Nikolova et al., 2006). Also during this time, the roots temporarily stopped growing which was not the case in previous years (i.e. 1999, 2000, and 2002) when a sufficient water supply was present (Nikolova et al., 2010). The high degree of suberization of the FR can reduce radial hydraulic conductivity and may act as a physical barrier to the movement of water/nutrients into the plant (Steudle, 2000). Development of more suberized and lignified roots in drying soils has been described in plants with limited access to water, e.g. desert plants (Nobel and Huang, 1992) as well as in *Quercus ilex* and *Vitis vinifera* (Brunner et al., 2015 and the references therein) and *Pinus halepensis* (Leshem, 1970) and may serve as a mechanism to decrease water loss from roots. Orlov (1957) similarly observed color progression in absorbing roots of spruce under natural conditions and related this to the senescence of the cortex. The present study is, to our knowledge, the first study to report a shift in color/suberization in spruce fine-roots in response to drought. Such adjustment of FR may reflect an earlier senescence of the spruce fine-roots in order to enhance C retention during harsh drought, thus supporting the “slow” ecological strategy (**Figure 6**).

### Physiological Traits

Beech had higher RR than spruce across all root types. In beech, this high RR was driven by FR, despite this category containing roots of a greater diameter compared to MR (e.g., in spring). This finding conflicts with other studies that report a decrease in RR with root diameter (e.g., Pregitzer et al., 1998; Di Iorio et al., 2016). Absorptive roots, like FR, represent primary roots with active cell divisions within the apical meristem, and a determinate growth pattern (Dubrovsky, 1997; Heimsch and Seago, 2008). Thus, the stimulation of root respiration and growth during the summer dry periods could be a result of phytohormonal control (Chapman et al., 2003; Perrot-Rechenmann, 2014) likely leading to temporary dysfunction of the primary-root apical meristem but stimulation of lateral-root production. Dubrovsky (1997) suggested the purpose of such dysfunction is to allow for the formation of lateral roots which quickly elongate (i.e. respire) and then slow down and finally cease elongation only a few days after emergence. In cactus, Dubrovsky (1997) related this adjustment to temporary water availability in arid environments (see also Chapman et al., 2003). In the present study, similar mechanisms may have induced a formation of lateral-like ephemeral FR roots with high SRA in beech. These young FR maintained the high RR of the beech rootlets during the rest of the growing season, thus representing a “fast” ecological strategy (**Figure 6**). Consequently, the formation of ephemeral root parts may represent a competitive advantage of beech, enabling rapid proliferation when resources are available (Nikolova, 2007; Bauerle, in prep.) or may play a role in hydraulic redistribution when present (Caldwell et al., 1998; Bauerle et al., 2008; Zapater et al., 2011).

During spring, spruce had the highest RR and *Q_10_* in FR. During late summer, when soils were extremely dry, spruce FR possessed the lowest RR but had the highest metabolic activity (as reflected by high *Q_10_*) among all three fine-root categories. The suberized exodermis protects the FR from desiccation (Cruz et al., 1992; Steudle, 2000), however at the price of C expenditure for respiration. Since the proportion of FR in the rootlet biomass was low (<10%) in spruce, this fine-root category did not drive the RR of rootlets for this species.

Overall, in both tree species, MR had medial RR, with the exception of spruce in spring and late summer, when MR had the highest respiration among all root categories. This could be explained by the relatively high percentage of non-mycorrhizal root tips (ca. 45%; Nikolova, 2007), which are fast-growing (Brundrett, 2002) and were produced in new root flushes during spring. In contrast, in late summer, the respiration activity of MR was likely needed to support the high fungal colonization rate (over 90%; Nikolova, 2007). In spruce rootlets, MR is likely driving the respiration activity during drought, thereby following a “fast” ecological strategy (**Figure 6**).

Not surprisingly, respiration was low in TR in both tree species, as such aged root sections (cf. Solly et al., 2018) with secondary growth serve water/nutrient transport rather than resource uptake (Lobet et al., 2014) indicating a “slow” ecological strategy. The lowest *Q_10_* was also measured in TR (around 1.10), as RR approached the level of maintenance respiration (RR^10^ around 2.5 nmol CO_2_ g^−1^s^−1^, in both tree species), in the absence of high temperature sensitivity (Burton et al., 1998).

### Carbon and N Concentrations

In beech rootlets, C concentration was the highest during early and late summer. This corresponds to the seasonal C trend reported for beech fine-roots from adult coppice forests in Italy’s Prealps (Terzaghi et al., 2013). In contrast, C concentration of spruce rootlets consistently increased through the growing season, reaching higher levels than beech rootlets by the end of the growing season. In both tree species, C dynamics of the rootlets seemed to depend on the proportion of TR, which was the root category with the highest C concentration.

Beech rootlets only had significantly higher N concentration compared to spruce in late summer, which possibly resulted from the enhanced proportion of ephemeral roots with highest N. In rootlets, our results did not support the traits described by Reich (2014) for the foliar economic spectrum, that species with “fast” strategies, i.e. beech, will have higher N concentration compared to the “slow”-species, i.e. spruce. In contrast, our findings are in line with Weemstra et al. (2017) who found that root traits do not necessarily correlate with leaf traits, in particular in species with more conservative root traits (i.e. with thick roots and long root lifespan) such as (evergreens) conifers. Concerning these tree species, the discrepancy in leaf and root traits may result from the confounding effect of mycorrhiza on the seasonality in water and soil resources uptake.

According to Gordon and Jackson (2000) the average C:N ratio of fine roots <2 mm is 43:1 across a broad range of ecosystems and biomes. In beech fine-roots, Terzaghi et al. (2013) reported a C:N ratio from 40:1 to 90:1. Both studies reported on values which are much higher than the C:N ratio from our study. This discrepancy occurred as a result of the higher N concentration we found in beech and spruce rootlets (13–19 mg g^−1^). This higher N concentration of rootlets in the trees from Kranzberger Forst is, however, not surprising, as it reflects the high levels of N deposition measured at that time in similar forests in Bavaria (20–25 kg ha^−1^ a^−1^; Raspe et al., 2018).

In our study, N concentration was a significant predictor for root RR of the rootlets (see also Burton et al., 1998; Ceccon et al., 2016), but the seasonal dynamics in these relationships was species-specific, in particular during the harsh drought in late summer. In beech, the N status varied largely within the rootlets, indicating, in this tree species, an adaptable fine-root system to a patchy soil environment. In contrast, spruce rootlets were more uniform and likely showed a temporal ‘dormancy’ during late summer, in response to increased soil moisture deficits. The differential N–RR relationship outlines contrasting coping strategies for beech and spruce in the presence of drought: Fast mobilization and use of internal C stores for new fine-root growth to ensure sustained resource uptake in beech, but reduced fine-root growth and uptake via suberization of FR in spruce to prevent resource loss.

### Non-Structural Carbohydrates

Non-structural carbohydrates are crucial in mitigating drought stress in plants (O’Brien et al., 2014; Hartmann and Trumbore, 2016; Hartmann et al., 2018). During drought, there is a distinct trade-off between growth and reserve accumulation, eventually leading to a decline of NSC concentrations in above-ground organs (McDowell, 2011) with simultaneously enhanced reallocation to roots (Brunner et al., 2015). Stored carbohydrates are important particularly in deciduous species that need to rely on the stored reserves to initiate leaf and root growth (Chapin et al., 1990; Landhäusser et al., 2012). In beech rootlets from our study, TSC increased through the growing season. This dynamic was largely driven by the sucrose content of the FR, which had the highest TSC reserves during late summer, to meet the C demand for respiration and regrowth. Physiologically active FR allow an increased water uptake and transfer to aboveground organs (Badri and Vivanco, 2009; Karst et al., 2016). TSC also remained high in transport fine-roots. The observed TSC accumulation in TR may lower the osmotic potential (Smirnoff, 1995; Brunner et al., 2015; Hommel et al., 2016), allowing for prolonged functionality of the aging roots responsible for transport and storage under drought. Such enhanced accumulation of assimilates reveals a “slow” ecological strategy of transport fine-roots in beech (**Figure 6**). In MR, sucrose, glucose and fructose concentrations almost doubled during the second half of the growing season, probably to maintain the high ectomycorrhizal colonization under drought (Shi et al., 2002).

In spruce rootlets, lowest levels of TSC were detected late in the growing season, corresponding to the enhanced proportion of MR with depleted sugars and starch concentration in autumn. In spruce FR and TR, highest TSC concentrations were found during the dry late summer, which is in line with temporally increased soluble sugars in roots in response to drought (McDowell, 2011; Müller et al., 2016). Thus, in both fine-root categories (FR and TR) a “slow” ecological strategy to drought adaptation was detected (**Figure 6**). Such temporal sugar accumulation may result from photosynthesis exceeding water-limited growth demands (Körner, 2003). Drought-induced TSC accumulation may be a common mechanism for survival during periods of stress in spruce. The high root concentrations of glucose, fructose, and pinitol may not only decrease the root water potential, facilitating water absorption from dry soil (Lambers et al., 1998) but also aid in sustaining and extending the mycelial network (Ekblad et al., 2013). For example, sustaining roots with a mycorrhizal association during drought requires sucrose to be hydrolyzed to glucose and fructose, and then consumed by both the fungal partners and root cortical cells (Nehls and Hampp, 2000). Although such use of sucrose is relevant for maintaining the respiration of MR in both tree species, spruce in particular, appears to rely more heavily on its mycorrhizae to withstand drought (Paradiso et al., 2019 and references therein). Under recurring prolonged episodes of drought, the persistence of drought-adapted ECM fungi can aid in tree survival, where trees with functional associations tolerating soil water potentials as low as −3 to −5.5 MPa (Smith and Read, 2008).

In this study, fine-root starch concentrations in both tree species were 10–100-fold lower than previously reported values for adult trees under natural growing conditions (e.g., Brunner et al., 2002; Barbaroux et al., 2003; Terzaghi et al., 2016; Rosinger et al., 2020). Soil water availability differed between the two tree species starting in the spring of 2003 (Nikolova et al., 2009), a direct result of the ability of spruce to take up and transpire water before beech flushed its leaves (Beier, 1998). This resulted in a longer period of exhausted soil water availability for spruce (i.e., 75 d in spruce vs. 45 d in beech). Experimental studies show starch concentration in roots to strongly decrease with the strength of stress (Thomas et al., 2002; Braun et al., 2004), which may result in higher mortality of the non-mycorrhizal fine roots, and/or in reduced fungal diversity of the mycorrhizal roots (Shi et al., 2002; Pena et al., 2010). In beech, the low starch concentration may have predetermined the short lifespan (Marshall and Waring, 1985; Nikolova, 2007) and, thus, the ephemeral character of beech fine roots during exceptional drought. In spruce, fine roots with reduced growth and low starch reserves may eventually represent a starch degradation into glucose, needed to support the mycorrhizal network. This would be reasonable as fungi use sugar alcohols such as arabitol and mannitol to enhance their osmotic strength during drought (Shi et al., 2002), and to enlarge their mycelium to proliferate into deeper and wetter mineral soil horizons (Ekblad et al., 2013). The heavy seed production that occurred for both tree species during 2003 (Dietrich et al., in prep.) could also have induced a major reallocation of the mobile C-pool into reproductive organs (Körner, 2003), additionally declining the stored starch in roots.

### Specifics of the Study

The present study was a part of a larger project, where a comprehensive investigation on the belowground effects of an experimentally enhanced ozone regime was conducted from 2002 through 2004 (Matyssek et al., 2010). While this study was not explicitly set-up to study species specific drought effects we were able to capture this response through several of our samples. The systematic measurement of root RR and other related parameters was planned only for 2003 (cv. Nikolova, 2007), and only short methodological checks on these parameters were done during the non-limited years 2002 and 2004. Nevertheless, other parameters recorded systematically from 2002 to 2004 such as the “autotrophic” and “heterotrophic” soil RR, the fine-root production and the fine-root recovery rate (Nikolova et al., 2009), as well as the total soil RR, the standing fine-root biomass, the amount of annually produced fine-root biomass and its δ^13^C signature (Nikolova et al., 2010) depicted contrasting responses of both species during 2003 with spruce being more effected by extreme drought compared to beech. This result is not surprising considering the different growth habits including leaf physiology, branching architecture (funnel-like in beech), rooting depth (shallow-rooted in spruce) of deciduous vs. evergreen species (Nikolova et al., 2009). But the underlying mechanisms of such species-specific adjustments to seasonal drought still remained unclear. Despite the limitation of not having reference data from “normal” conditions, the present study makes use 1/100 (1 in 100 years) drought event to analyze the belowground adaptability of these two most important Central European tree species (Ellenberg, 1996). Our investigation presents novel seasonal data on a broad range of fine-root traits and suggests possible mechanisms of adaptability, which should be tested in further experiments on adult forest trees.

## Conclusions

We found for fine-roots in beech and spruce, that each species is not tied to one sole ecological strategy in coping with drought. In beech, the youngest absorptive FR followed the “fast” strategy, i.e. short-lived roots, enlarged specific fine-root area, and high root RR. High ECM colonization typical of mycorrhizal roots also represents a “fast” ecological strategy. These adjustments indicated enhanced C turnover, which facilitate effective acquisition of available belowground resources and rapid translocation of resources to aboveground organs. Transport fine-roots with developed secondary xylem, however, followed a “slow” strategy, as NSC increased during drought, possibly preventing resource efflux and root desiccation. Overall, during seasonal drought, beech fine-root traits largely reflected a “fast” strategy, particularly the youngest absorptive fine-roots.

In contrast, fine-root traits of spruce reflected largely a “slow” strategy. Suberized foraging fine-roots and the transport fine-roots had larger diameters and higher NSC levels facilitating long-term C retention during drought. Such adjustments can protect spruce fine-roots against desiccation and lower C required for respiration. However, absorptive mycorrhizal fine-roots were more indicative of the “fast” strategy. Therefore, the resource acquisition in spruce during drought seems to largely rely on mycorrhizal fungi.

The present study took advantage of the naturally occurring severe drought during the summer of 2003. However, the question remains as to what extent do beech and spruce trees recover from successive years of drought. Such scenarios are realistic considering the recent droughts of 2018 and 2019 in Central Europe. Our findings indicate that beech trees could likely suffer from C starvation during successive drought events since root regeneration can deplete the C reserves under drought-impaired photosynthesis. Spruce’s survival lies heavily in the ability of mycorrhizal communities to survive recurring drought and continue to contribute to water/nutrient uptake and plant vitality. If instead the mycorrhizal association becomes a competitor during drought (Kariman et al., 2018) and therefore a dwindling C supply in weakened trees then the fate of spruce is also likely dire.

## Data Availability Statement

The raw data supporting the conclusions of this article will be made available by the authors, without undue reservation.

## Author Contributions

PN, IB, RM, K-HH, and HB conceptualized the main research questions. PN collected data and performed the data analyses. PN wrote the manuscript, RM, TB, K-HH, HB, and IB revised the manuscript.

## Funding

This work was funded by the Deutsche Forschungsgemeinschaft (DFG), through SFB 607 “Growth and Parasite Defence—Competition for Resources in Economic Plants from Agronomy and Forestry”.

## Conflict of Interest

The authors declare that the research was conducted in the absence of any commercial or financial relationships that could be construed as a potential conflict of interest.

The reviewer IB declared a past co-authorship with one of the authors [IB] to the handling editor.
