# Supplementary material for: Fine-Root Traits Reveal Contrasting Ecological Strategies in European Beech and Norway Spruce During Extreme Drought
Source: Front Plant Sci. 2020 Aug 13;11:1211. doi: 10.3389/fpls.2020.01211 (PMC7438540; doi:10.3389/fpls.2020.01211)
Supplement: Supplementary file 1 [file DataSheet_1.pdf]

Supplementary Material

1.1 Supplementary Figures

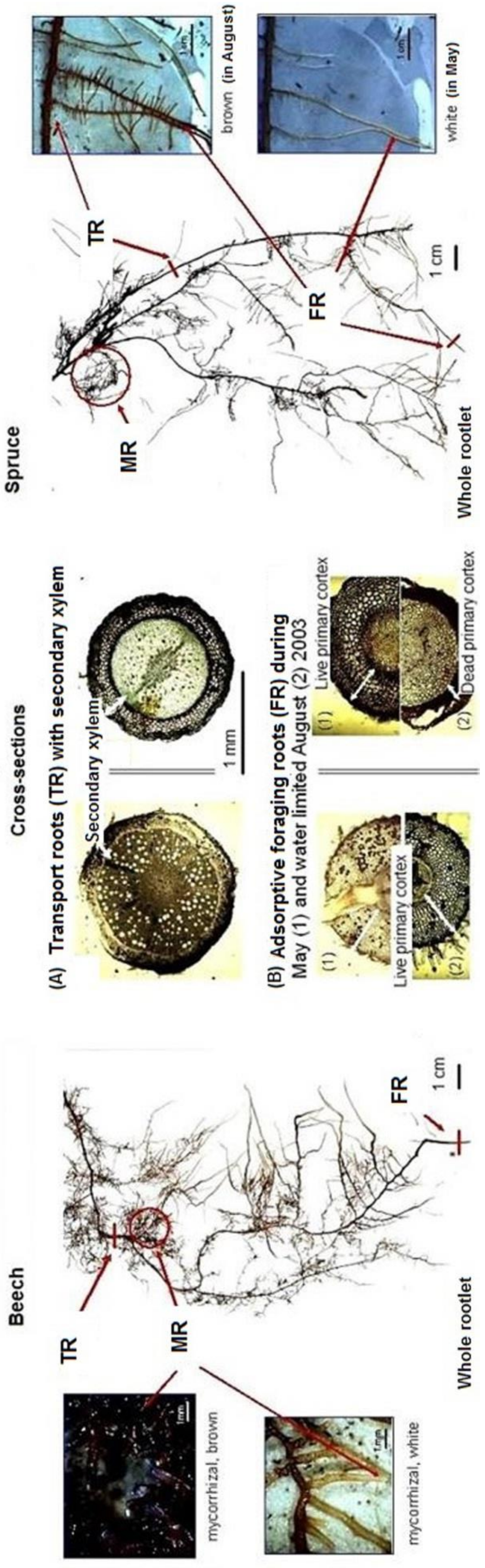

Supplementary Figure S1. Exemplifying rootlet classifications in beech (left) and spruce (right). Red lines denote cross-sectional positions related to FR = absorptive foraging roots with primary xylem and TR = transport roots with secondary xylem, and red circles such of proliferating MR = absorptive mycorrhizal fine roots. Cross-sections of TR (A) and FR (B) from May/spring (1) and August/late summer (2) of 2003.

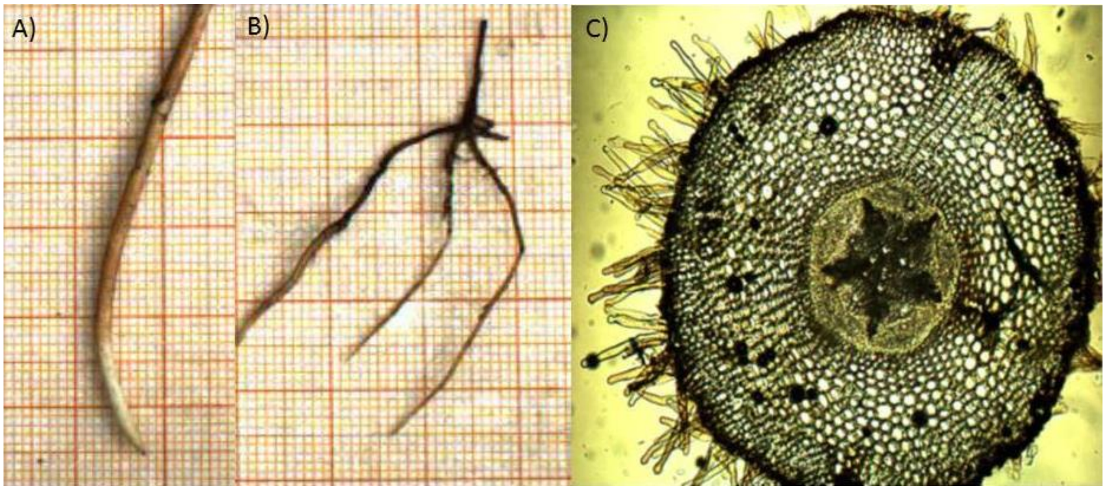

Supplementary Figure S2. Foraging roots in beech during May 2003/spring (A), and August/late summer 2003 (B). Image (C) exemplifies the transverse section typical for both, (A) and (B) roots.

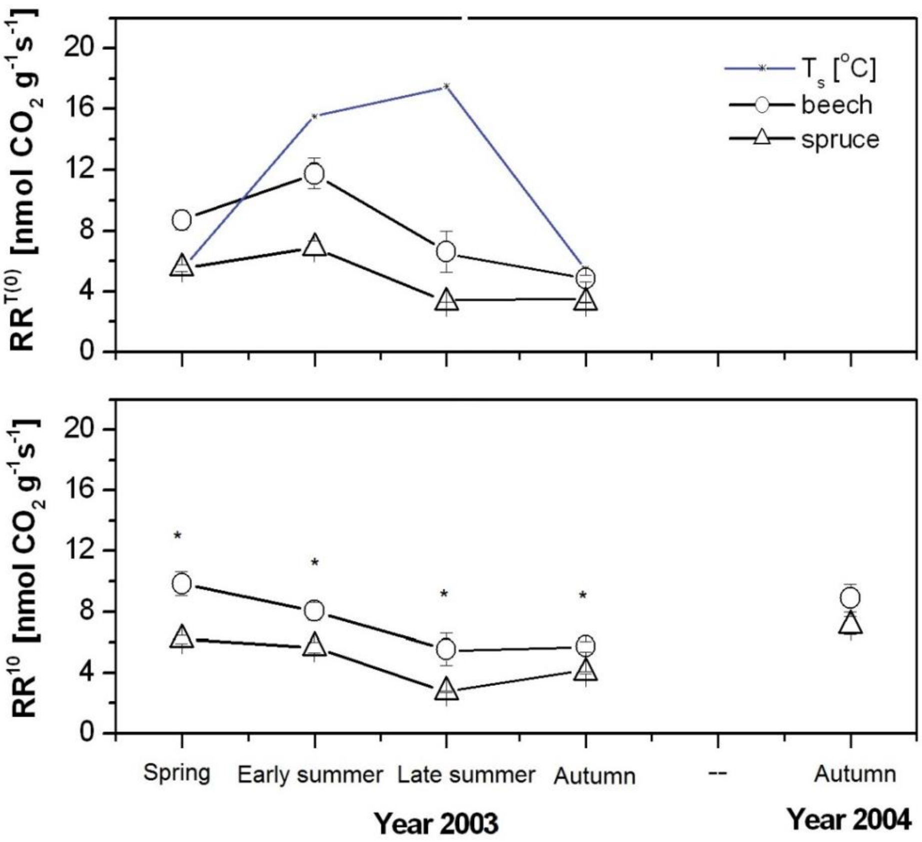

Supplementary Figure S3. Respiration rates of individual rootlets in beech and spruce. The upper graph represents respiration rates $RR^{T(0)}$ at mean daily soil temperature T_s (monitored at 0 cm soil depth); the lower graph shows respiration rates RR^{10} at a standard T = 10°C. Asterisks represent significant differences in RR^{10} between beech and spruce (*U*-test, $p < 0.05$). Means ± 95 % confidence interval (n = 7 to 10).

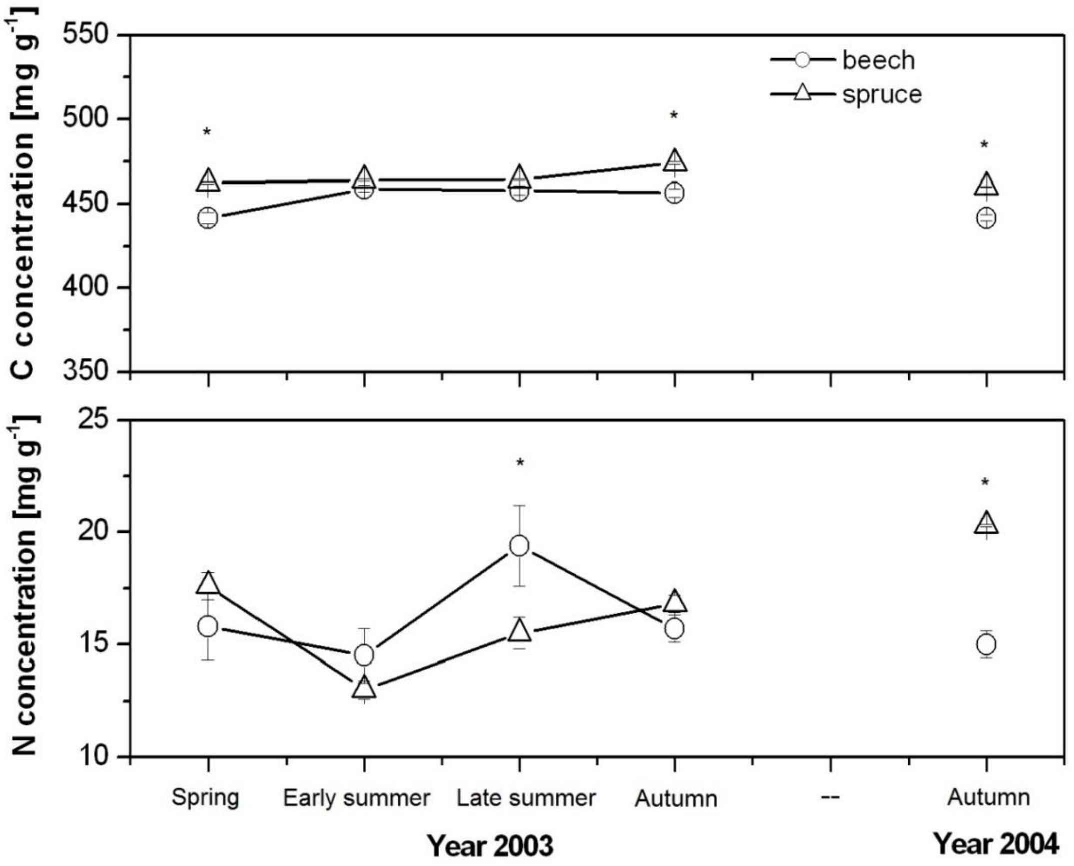

Supplementary Figure S4. Carbon (C) and nitrogen (N) concentration in individual rootlets of beech and spruce. Asterisks represent significant differences between beech and spruce (*U*-test, $p < 0.05$). Means ± 95% confidence interval (n = 7 to 10).

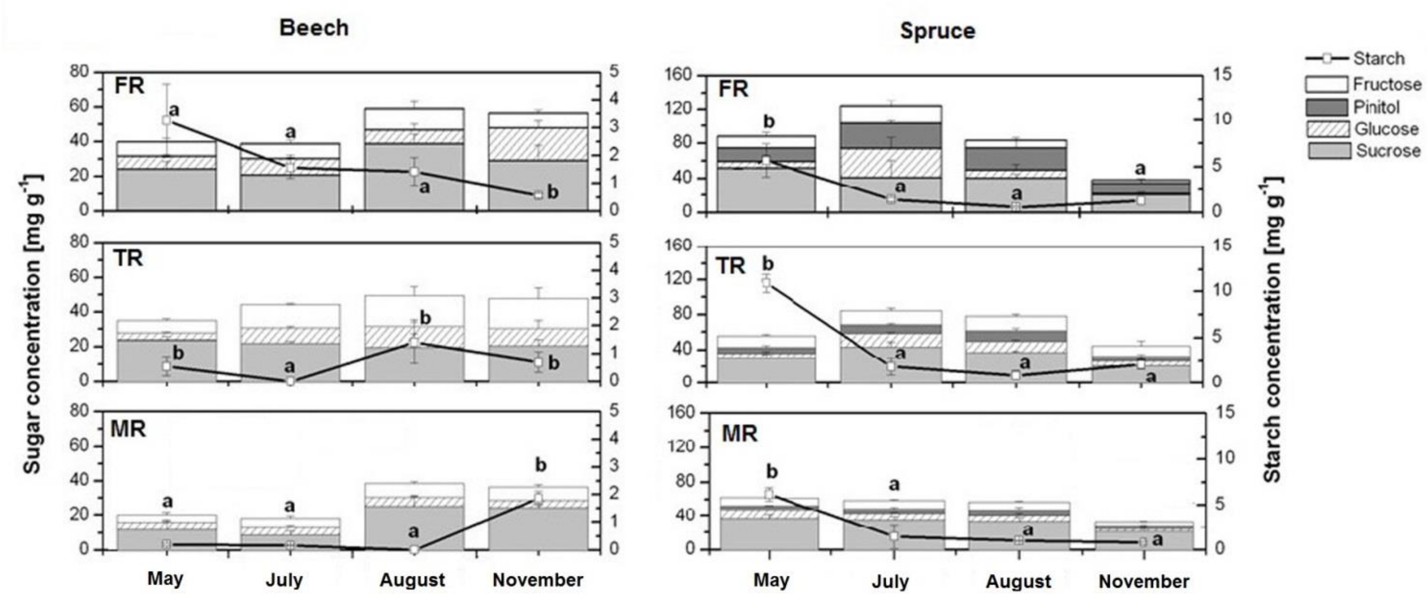

Supplementary Figure S5: Seasonal course of carbohydrate fractions during 2003 in the adsorptive foraging fine-roots FR, transport fine-roots TR and adsorptive mycorrhizal fine-roots MR in beech and spruce. TSC = total sugar content. Within each species and fine-root category, significant differences in starch concentration between sampling campaigns are indicated by different letters (ANOVAs with Tukey-HSD post hoc tests, $p < 0.05$). Data shown as means ± 95% confidence interval (n = 4 to 10).

1.2 Supplementary Tables

Supplementary Table S1. Dry weights DW [g] and proportions [%] of three fine-root categories in the biomass of eight individual beech rootlets (b1 to b8): FR = absorptive foraging roots with primary xylem, TR = transport roots with secondary xylem, MR = absorptive mycorrhizal fine roots. Average proportions of each of the three fine-root categories in total rootlet biomass are shown in italics (means ± 1 standard error).

Sampling campaign	Individual rootlet	Dry weight [g]				Proportion [%]		
		FR	TR	MR	Rootlet	FR	TR	MR
Spring	b1	0.149	0.785	0.473	1.407	10.6	55.8	33.6
	b2	0.129	0.636	0.432	1.197	10.7	53.1	36.1
	b3	0.070	0.134	0.195	0.399	17.5	33.6	49.0
	b4	0.150	0.169	0.093	0.412	36.4	41.1	22.5
	b5	0.148	0.465	0.465	1.077	13.7	43.1	43.2
	b6	0.133	0.240	0.202	0.575	23.1	41.7	35.2
	b7	0.133	0.104	0.129	0.367	36.3	28.4	35.3
	Mean ± SE					*21.2 ± 4.2*	*42.4 ± 3.7*	*36.4 ± 3.1*
Early summer	b1	0.115	0.164	0.136	0.415	27.7	39.6	32.7
	b2	0.351	0.302	0.389	1.042	33.7	29.0	37.3
	b3	0.306	0.113	0.272	0.691	44.3	16.4	39.4
	b4	0.070	0.178	0.333	0.581	12.0	30.7	57.3
	b5	0.105	0.058	0.091	0.254	41.5	22.8	35.8
	b6	0.108	0.099	0.232	0.439	24.5	22.5	53.0
	b7	0.137	0.232	0.240	0.609	22.5	38.1	39.4
	b8	0.040	0.064	0.091	0.195	20.5	32.8	46.7
	Mean ± SE					*28.4 ± 4.2*	*27.5 ± 2.6*	*44.1 ± 3.0*
Late summer	b1	0.478	0.807	0.320	1.605	29.8	50.3	19.9
	b2	0.121	0.195	0.287	0.603	20.1	32.3	47.6
	b3	0.224	0.024	0.313	0.561	40.0	4.2	55.8
	b4	0.667	0.548	0.667	1.882	35.4	29.1	35.4
	b5	0.308	0.278	0.437	1.023	30.1	27.2	42.7
	b6	0.299	0.040	0.231	0.570	52.4	7.0	40.5
	b7	0.183	0.042	0.283	0.508	36.0	8.3	55.7
	b8	0.577	0.557	0.614	1.748	33.0	31.9	35.1
	Mean ± SE					*35.3 ± 3.5*	*20.0 ± 4.5*	*44.7 ± 3.1*
Autumn	b1	0.030	0.117	0.123	0.270	11.1	43.3	45.6
	b2	0.145	0.285	0.215	0.645	22.5	44.2	33.3
	b3	0.080	0.197	0.142	0.418	19.1	47.0	33.9
	b4	0.135	0.361	0.350	0.847	15.9	42.7	41.4
	b5	0.097	0.225	0.153	0.475	20.4	47.3	32.2
	b6	0.090	0.447	0.507	1.043	8.6	42.8	48.6
	b7	0.042	0.367	0.520	0.929	4.6	39.5	56.0
	b8	0.163	0.175	0.289	0.627	26.0	27.9	46.1
	Mean ± SE					*16.7 ± 2.7*	*41.7 ± 2.3*	*41.6 ± 3.2*

Supplementary Table S2. Dry weights DW [g] and proportions [%] of each of the three fine-root categories in the biomass of ten individual spruce rootlets (s1 to s10): FR = absorptive foraging roots with primary xylem, TR = transport roots with secondary xylem, MR = absorptive mycorrhizal fine roots. Averaged proportions of each of the three fine-root categories in total rootlet biomass in italics (means ± 1 standard error).

Sampling campaign	Individual rootlet	Dry weight [g]				Proportion [%]		
		FR	TR	MR	Rootlet	FR	TR	MR
Spring	s1	0.159	1.169	0.578	1.906	8.4	61.3	30.3
	s2	0.117	1.148	0.756	2.021	5.8	56.8	37.4
	s3	0.114	3.830	1.480	5.424	2.1	70.6	27.3
	s4	0.027	0.628	0.592	1.248	2.2	50.4	47.4
	s5	0.024	0.758	0.800	1.581	1.5	47.9	50.6
	s6	0.093	0.798	0.722	1.613	5.8	49.5	44.8
	s7	0.078	0.343	0.311	0.732	10.6	46.9	42.5
	s8	0.084	0.425	0.280	0.789	10.6	53.9	35.5
	s9	0.088	0.505	0.362	0.954	9.2	52.9	37.9
	Mean ± SE					*6.0 ± 1.4*	*53.2 ± 2.9*	*40.8 ± 2.8*
Early summer	s1	0.000	1.329	0.522	1.852	0.0	71.8	28.2
	s2	0.050	0.865	0.831	1.746	2.9	49.5	47.6
	s3	0.124	1.485	1.262	2.871	4.3	51.7	44.0
	s4	0.000	0.808	0.457	1.265	0.0	63.9	36.1
	s5	0.000	0.443	0.310	0.753	0.0	58.8	41.2
	s6	0.123	0.569	0.758	1.450	8.5	39.3	52.3
	s7	0.087	0.559	0.862	1.508	5.8	37.0	57.2
	s8	0.018	0.849	0.282	1.149	1.6	73.8	24.6
	Mean ± SE					*3.3 ± 1.1*	*53.4 ± 4.7*	*43.3 ± 3.8*
Late summer	s1	0.051	0.608	0.527	1.186	4.3	51.3	44.4
	s2	0.066	0.366	0.197	0.629	10.5	58.2	31.3
	s3	0.000	0.334	0.583	0.917	0.0	36.4	63.6
	s4	0.276	0.815	0.406	1.497	18.4	54.4	27.1
	s5	0.081	0.581	0.995	1.657	4.9	35.1	60.0
	s6	0.041	0.456	0.297	0.794	5.2	57.4	37.4
	s7	0.028	0.149	0.135	0.312	9.0	47.8	43.3
	s8	0.043	0.209	0.207	0.459	9.4	45.5	45.1
	s9	0.180	0.327	0.542	1.049	17.1	31.2	51.7
	s10	0.015	0.299	0.189	0.503	3.0	59.4	37.6
	Mean ± SE					*9.5 ± 1.9*	*47.3 ± 3.5*	*43.2 ± 3.4*
Autumn	s1	0.050	0.274	0.323	0.648	7.8	42.3	49.9
	s2	0.047	0.401	0.642	1.090	4.3	36.8	58.9
	s3	0.000	0.954	1.303	2.257	0.0	42.3	57.7
	s4	0.038	0.538	0.967	1.543	2.4	34.9	62.7
	s5	0.063	0.744	1.023	1.829	3.4	40.7	55.9
	s6	0.081	0.676	0.965	1.721	4.7	39.3	56.0
	s7	0.024	0.627	0.784	1.435	1.7	43.7	54.6
	s8	0.000	0.182	0.881	1.063	0.0	17.1	82.9
	s9	0. 025	0.568	0.655	1.482	1.7	38.3	60.0
	Mean ± SE					*2.9 ± 0.8*	*37.2 ± 2.5*	*59.8 ± 2.9*
